# External validation of an artificial intelligence multi-label deep learning model capable of ankle fracture classification

**DOI:** 10.1186/s12891-024-07884-2

**Published:** 2024-10-04

**Authors:** Jakub Olczak, Jasper Prijs, Frank IJpma, Fredrik Wallin, Ehsan Akbarian, Job Doornberg, Max Gordon

**Affiliations:** 1 Danderyd University Hospital, Karolinska Institute, Stockholm, Sweden; 2 Flinders University and Medical Centre, Adelaide, South Australia Australia; 3 University Medical Center Groningen, University of Groningen, Groningen, Netherlands

**Keywords:** External validation, Machine learning, Neural networks, Ankle, Trauma, AO/OTA classification

## Abstract

**Background:**

Advances in medical imaging have made it possible to classify ankle fractures using Artificial Intelligence (AI). Recent studies have demonstrated good internal validity for machine learning algorithms using the AO/OTA 2018 classification. This study aimed to externally validate one such model for ankle fracture classification and ways to improve external validity.

**Methods:**

In this retrospective observation study, we trained a deep-learning neural network (7,500 ankle studies) to classify traumatic malleolar fractures according to the AO/OTA classification. Our internal validation dataset (IVD) contained 409 studies collected from Danderyd Hospital in Stockholm, Sweden, between 2002 and 2016. The external validation dataset (EVD) contained 399 studies collected from Flinders Medical Centre, Adelaide, Australia, between 2016 and 2020. Our primary outcome measures were the area under the receiver operating characteristic (AUC) and the area under the precision-recall curve (AUPR) for fracture classification of AO/OTA malleolar (44) fractures. Secondary outcomes were performance on other fractures visible on ankle radiographs and inter-observer reliability of reviewers.

**Results:**

Compared to the weighted mean AUC (wAUC) 0.86 (95%CI 0.82–0.89) for fracture detection in the EVD, the network attained wAUC 0.95 (95%CI 0.94–0.97) for the IVD. The area under the precision-recall curve (AUPR) was 0.93 vs. 0.96. The wAUC for individual outcomes (type 44A-C, group 44A1-C3, and subgroup 44A1.1-C3.3) was 0.82 for the EVD and 0.93 for the IVD. The weighted mean AUPR (wAUPR) was 0.59 vs 0.63. Throughout, the performance was superior to that of a random classifier for the EVD.

**Conclusion:**

Although the two datasets had considerable differences, the model transferred well to the EVD and the alternative clinical scenario it represents. The direct clinical implications of this study are that algorithms developed elsewhere need local validation and that discrepancies can be rectified using targeted training. In a wider sense, we believe this opens up possibilities for building advanced treatment recommendations based on exact fracture types that are more objective than current clinical decisions, often influenced by who is present during rounds.

**Supplementary Information:**

The online version contains supplementary material available at 10.1186/s12891-024-07884-2.

## Introduction

With artificial intelligence's (AI) growing success in image analysis, AI interventions are rapidly being developed and applied in medical diagnostics [1]. Many studies have reported promising results, reaching close to perfect accuracy on basic pathology detection tasks, illustrating that accuracy in elementary pathology detection should be relatively easy to attain. The promise of AI interventions lies in their ability to solve complex tasks in different scenarios. For example, classifying fractures into meaningful features could give clinical guidance, drive treatment decision-making, or predict clinical outcomes. However, as researchers develop models under controlled conditions, few have reproduced their results.

A meta-analysis by Liu et al. reported the lack of external validation in deep learning [2]. For example, a systematic meta-analysis by Oliveira e Carmo et al. found 36 papers using deep learning for orthopedics. Only three were externally validated, i.e., tested on independent data from a different site [3–6] (See Supplement 1, Table S1.) Similarly, a systematic review of orthopedic machine learning models predicting surgery outcomes by Groot et al. found that only 10/59 studies had externally validated their models [7]. There are currently many initiatives to improve the quality of reporting AI studies in medicine, for example, via checklists for consistent and relevant reporting and external validation [1, 8]. For a predictive model to be helpful, it must work and be tested in clinical environments other than what the model has been trained on – also called external validation – and thus be generally applicable.

There are three major classification systems for ankle fractures. Previously, we showed that deep-learning models can classify ankle fractures according to the AO Foundation/Orthopedic Trauma Association (AO/OTA) classification. The AO/OTA standard classifies fractures based on their visual appearance in radiographic examinations, making it well-suited to AI image classification. This classification is influenced by the very popular Lauge-Hansen (LH) system, which is widely used in clinical practice and categorizes fractures based on the injury mechanism. The LH system's reliance on such non-visual factors presents challenges for this study, where the injury mechanisms were missing. At the same time, the AO/OTA classification can be seen as an extension of the Danis-Weber classification.

We have previously reached a model performance of weighted average area under the receiver operating characteristic curve (AUC and wAUC) 0.90 (95%CI 0.82–0.94)[9] using internal validation test data – data from the same site as the test data. Given that such a model aims to facilitate classification and decision-making in an emergency setting, we needed to validate its performance in the clinic, not in the training setting. This paper examines the external validation of an AI model for classifying ankle fractures according to the AO/OTA standard. External validation consists of applying a model to independent data from a site different from the one used for training. It aims to see how relevant and generalizable a model is in a clinical context. Our primary aim was to study the effect of transferring a model to a different setting, i.e., the model’s external validity, and to study ways to improve the external validity of a machine learning model. Our secondary aim was to explore the AO/OTA classification more broadly.

## Material and methods

The study was a retrospective external validation cohort study.

### Ethics approval and consent to participate

Ethical approval for the collection of Flinders/external validation dataset was obtained from the Central Adelaide Local Health Network Human Research Ethics Committee (CALHN HREC) reference number: 13991, Authorization date: 21 December 2020. In accordance with the ethical permit, no individual or informed consent from participants was required. In accordance with the specific consent for inclusion into this study, the data was not considered patient data.

Ethical approval for the Danderyd/internal validation dataset was obtained by the Regional Ethics Committee for Stockholm, Sweden (Dnr. 2014/453-31/3, April 9, 2014). According to the ethical approval, no individual or informed consent from participants was required, as the data did not constitute human data after anonymous collection.

Ethical approval to use the external dataset for this study was also obtained from the Swedish Ethical Review Authority, Sweden (Dnr. 2023-07151-01).

The need for informed consent for the use of the data for the study waived. The data was anonymous radiographs without personal identifiable information, it would not be possible to identify individuals and informed consent was waived.

### Data collection and pre-processing

#### Training and internal validation dataset (IVD)

The training data came from a retrospective cohort of trauma radiographs (initial imaging performed at the emergency department at the time of injury) collected from Danderyd University Hospital (Stockholm, Sweden) between 2002 and 2015. This level 2 trauma center had a referral area of approximately 350,000 people during that period. The data was collected anonymously and only coded with a unique patient identifier, but the radiologist report was included. No injury or population data (such as age, gender, trauma mechanism, etc.) was collected. We used the radiologist reports to generate initial fracture/no fracture labels. These labels have been improved over successive studies through manual review by radiologists and orthopedic consultants [9–11]. All examinations visualizing the ankle were included, and only pediatric studies (open physis) were excluded because they are classified differently.

Four hundred patients (409 exams, including all available views visualizing the ankle) were previously selected for the internal validation dataset (IVD). Our previous study had a 2/3 bias towards fractures in the IVD to ensure sufficient fractures to classify and compare rarer fractures. We did not specify the fracture type, so tibia, malleolus, fibula, or foot fractures were included [9, 10]. As part of active training, we added 2664 fractures to the training dataset to align it more with the EVD distribution. We used model-based selection, i.e., the model screened ankle studies from the Danderyd dataset and selected cases where the model flagged categories of interest or where the probability for the predicted class was low, i.e., had high uncertainty. These were then manually reclassified.

**Fig. 1:**
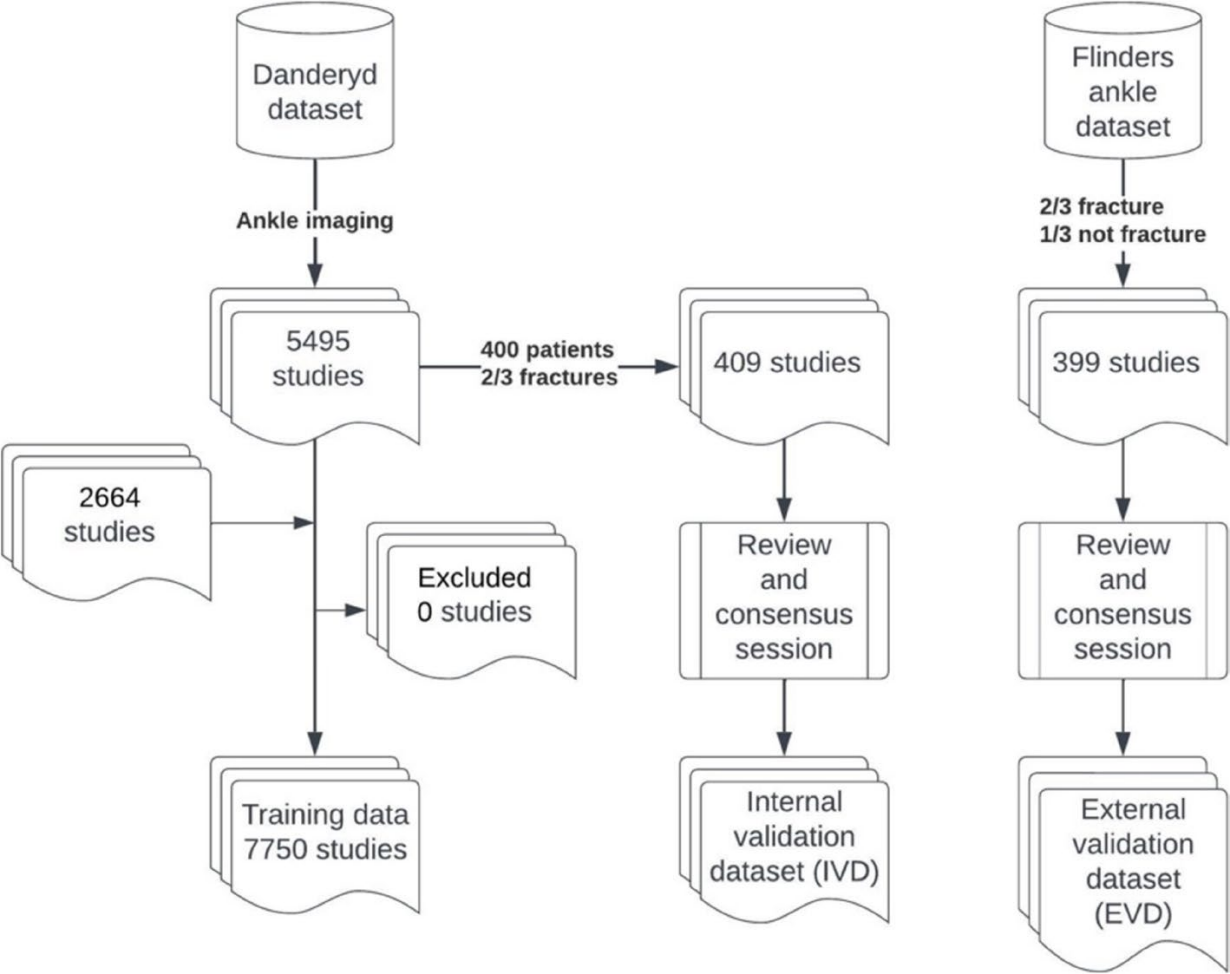
Study flowchart

No patient was present in both the training and IVD set. See Fig. 1. For training details, see Supplement 2.

#### External validation dataset (EVD)

The external validation dataset (EVD) was a subset of 12,000 radiographic ankle examinations collected from Flinders University Medical Centre (Adelaide, Australia), a level 1 Trauma Centre, between 2016 and 2020.

Studies were filtered using keywords in radiology reports to create an index database containing isolated fibular and lateral malleolus fractures and a non-fracture database.

While only trauma radiographs were included, this included one-week follow-ups and weight-bearing images. Projections were three standard views (AP, mortise, and lateral). Exclusion criteria were any pathology other than a fibula or lateral malleolus fracture, old fractures, callous or cast presence, radiographs of poor quality, open physes, radiological views of insufficient quality, and the occurrence of plates or screws were also excluded.

Three hundred ninety-nine examinations were randomly selected from the Flinders dataset and provided as an external validation dataset (EVD) for this study, with a 2/3 selection bias towards studies containing a fracture. The Flinders set was anonymized and provided without reports, injury, or population parameters [12] (Table 1.)

**Table 1:** Properties of the internal validation dataset (IVD) and external validation dataset (EVD)

Dataset properties	IVD		EVD	
Cases		409		399
Projections		≥ 4		3
Focus	Ankle study		Lateral malleolar fracture	
Timing	Initial imaging		Initial imaging, one-week follow-up, weight-bearing	
Implants & casts		Yes		No
Open physes		No		No
Excluded on imaging quality		None	Insufficient quality views Poor quality images Severely displaced fractures	
**Fracture**	**Cases**	**Percent (%)**	**Cases**	**Percent (%)**
Base	253	61,9%	277	69,4%
Malleolar^a^	216	52,8%	274	68,7%
Fibula^b^	37	9,0%	3	0,8%
Previous fracture/other^b^	*134*	*32,8%*	*15*	*3,8%*
Foot^b^	*57*	*13,9%*	*2*	*0,5%*

Numbers are based on ground truth labelling by reviewers after the consensus session

^a^Distinguishing between isolated fibula and lateral malleolar fracture can be subjective. In the absence of talar dislocation, we reviewed the radiologist’s report for indications of direct trauma. Additionally, we assessed for specific characteristics, such as the presence of more transverse fractures, which are more common in isolated fibula fractures, as opposed to C-category fractures that often extend beyond 3 cm. While these criteria are not entirely objective, we aimed to apply them consistently across cases to minimize variability

^b^Denotes fractures and outcomes that were flagged as fractures during study selection but are secondary outcomes

#### Annotation protocol for the EVD

The four reviewers (FIJ, EA, JD, and MG) were consultant or senior consultant orthopedic trauma surgeons. All underwent a training session to ensure familiarity with the labeling platform (the Raiddex platform developed by DeepMed AB) and agreement on the AO/OTA 2018 ankle fracture classification. Each reviewer labeled the EVD independently at the original image resolution. Labeling was distributed so that three reviewers examined each study independently. After the independent labeling, we held a consensus session to review the cases where there were discrepancies between observers, and a majority vote decided the final classification. The result was the ground truth EVD. We have previously established the annotations for the training data and the IVD ground truth [9].

### Model and evaluation

Image pre-processing, network architecture (modified ResNet-based [13] neural network model developed in PyTorch), parameters, training, and output evaluation were consistent with Olczak et al. 2021 [1] and identical for the IVD and the EVD. The network scaled down exams to reduced-size images for training and assessment. The network was always trained for 300 epochs, and we did not stop early.

The software used in our previous study was unsuitable for this study's experiments. Instead, we used an identically trained network on the same IVD. Due to the random nature of model training, the exact performance for the initial IVD varied slightly from our previous study. After initial evaluation, we were dissatisfied with the model performance and noted a notable difference in the distribution of Type A fractures. As part of active learning, we: 1) expanded the training data with previously unlabeled ankle imaging from the training site, focusing on type A fracture. These were labeled by FW (medical student) and JO (medical doctor). We could not preselect fracture type among those previously unclassified images. 2) we actively focused on Type A fracture prediction edge cases. Fractures in the training set classified as Type A with the lowest probability or where type A fracture was the second most likely type (but another type won out) were rigorously reexamined after each training epoch by MG (senior orthopedic consultant). By focusing on the lowest probability type A fractures and almost type A, we hoped to reduce the uncertainty in the type A classes. In addition to adding more training data, we trained the model on different image resolutions. We report the results for the 400 × 400-sized images as the primary outcome. At higher resolutions, there was no performance increase.

The model classified studies by examining all available images individually and independently for each possible class. There were 39 outcomes for ankle (AO/OTA 44) fractures and, as many classes for fibula (4), tibial (43), foot fractures, and one additional for fracture – yes/no. The model selected the most probable AO/OTA class (top-1 classification) for the series outcome [9]. Class outcomes, i.e., fracture yes/no, type (A–C – i.e., Danis-Weber), group (A1–C3), and subgroup (A1.1–C3.3) are determined independently of each other. We trained a network without pre-training, then used the resulting trained AI model to classify the IVD and EVD and compared the results to the ground truths. See Supplement 2 for details on the network, modeling parameters, and all possible outcomes for the network. We report our findings per the CAIR checklist [1] and follow the TRIPOD statement [14].

### Statistical analysis

#### Primary outcomes

The area under the receiver operating-characteristic curve (AUC) and the area under the precision-recall curve (AUPR) for malleolar fractures (AO/OTA 2018 bone-location 44). Top-1 classification is used for determining outcomes for each level, i.e., fracture/ vs. no fracture, type 44A-C, group 44A1-44C3, and subgroup 44A1.1-44A3.3, i.e., 40 possible outcomes for ankle fractures. While some outcomes overlap, each was decided individually. We used frequency-weighted means as summary statistics [1] and calculated 95% confidence intervals (CI) with bootstrapping. We do not report outcomes with single cases, as it is impossible to calculate CI for these outcomes.

Although AUC and accuracy are often used to report performance in CNN models, a multi-label classifier — such as that used in this study — benefits from a metric that can more accurately capture its inherent class imbalance between the many groups.

For the AUPR, a random classifier will perform proportionally to the number of positive outcomes for that class, i.e., *AUPR*_*random*_ = *(number of cases for the class/total number of cases)*. If a dataset consists of 10% of class *X*, a random classifier should deliver an AUPR of 0.1 for class *X*, and anything above that is better than chance [15]. Therefore, we also report when the AUPR outperforms a random classifier – i.e. when the lower 95%CI bound is better than the random classification. We only measured the top-1 performance (i.e., no points for being close to the correct answer).

After enhancing training (with active learning, additional training data, and increased image resolution), we only tested the model (on the EVD and IVD) once for each resolution. This was done to eliminate the risk of overfitting the EVD.

We compare the classifier's performance on both datasets and report according to the Clinical AI Research (CAIR) and TRIPOD checklists.

#### Secondary outcomes

Compare the classification between observers (before the consensus session) and performance for non-malleolar fracture outcomes. We use Cohen's kappa to compare two reviewers and intra-class correlation (ICC) to compare all reviewers. We use ICC and kappa as rough indicators of the difficulty of the classification task.

## Results

Compared to the IVD, the EVD had fewer displaced fractures and no casts or implants. The EVD included studies labeled "weight-bearing," indicating that these were not fresh injuries at the time of examination (i.e., from the emergency department at the time of injury). The EVD had three views per study, while the IVD had four or more views. The IVD had 216 ankle fractures out of 409 cases (53%), compared to 274 out of 399 ankle fractures (69%) in the EVD set (Table 1). The fracture incidence was similar, and type B fractures dominated both settings. Type A fractures were three times more prevalent in the EVD. There were more non-malleolar fractures in the IVD than in the EVD (Table 2 and Table 3). The EVD also had less severe fractures, e.g., more B1 fractures, less B3, and very few fibula fractures.

**Table 2:** Prediction outcomes for the internal validation set (IVD)

DANDERYD – Internal validation set (IVD) (409 cases)						
**Malleolar fractures**						
	**Cases**	**AUC (95% CI)**	**ΔAUC**	**AUPR (95% CI)**		**ΔAUPR**
Fracture	216	0.95 (0.94–0.97)	0.03	0.96 (0.94–0.97)^a^		0.03
**44A**						
Base	32	0.84 (0.76–0.92)	0.04	0.46 (0.11–0.35)^a^		0.23
44A1	22	0.84 (0.76–0.92)	-0.03	0.37 (0.08–0.29)^a^		0.19
44A1.1	6	0.88 (0.79–0.97)	-0.01	0.04 (0.01–0.10)		0.00
44A1.2	7	0.84 (0.69–1.00)	-0.02	0.30 (0.01–0.21)		0.22
44A1.3	9	0.82 (0.69–0.96)	0.03	0.18 (0.01–0.22)		0.11
44A2	7	0.99 (0.97–1.00)	0.15	0.52 (0.01–0.47)		0.28
44A2.1	5	0.99 (0.97–1.00)	0.09	0.41 (0.00–0.56)		0.15
44A2.3	2	0.99 (0.99–1.00)	0.14	0.25 (0.00–0.04)		0.23
44A3	2	0.95 (0.86–1.04)	-0.02	0.08 (0.03–0.17)^a^		0.01
**44B**						
Base	137	0.96 (0.93–0.92)	0.04	0.92 (0.88–0.95)^a^		0.05
44B1	67	0.95 (0.93–0.98)	0.05	0.77 (0.67–0.86)^a^		0.14
44B1.1	39	0.90 (0.87–0.94)	0.07	0.37 (0.25–0.51)^a^		0.06
44B1.2	26	0.94 (0.91–0.97)	0.07	0.40 (0.22–0.60)^a^		0.15
44B1.3	2	0.96 (0.90–1.02)	0.04	0.06 (0.01–0.23)^a^		0.03
44B2	38	0.86 (0.80–0.92)	0.01	0.40 (0.25–0.56)^a^		0.04
44B2.1	20	0.91 (0.85–0.97)	0.05	0.37 (0.20–0.55)^a^		0.14
44B2.2	16	0.88 (0.77–1.00)	-0.01	0.35 (0.15–0.53)^a^		0.13
44B2.3	2	0.87 (0.68–1.07)	-0.05	0.03 (0.00–0.11)^a^		0.00
44B3	32	0.92 (0.89–0.96)	0.06	0.50 (0.27–0.59)^a^		0.03
44B3.1	12	0.90 (0.83–0.97)	0.04	0.18 (0.06–0.34)^a^		0.02
44B3.2	13	0.92 (0.88–0.96)	0.08	0.20 (0.08–0.35)^a^		-0.04
44B3.3	6	0.96 (0.93–0.99)	0.02	0.16 (0.03–0.30)^a^		0.06
**44C**						
Base	47	0.93 (0.89–0.97)	0.05	0.73 (0.61–0.82)^a^		0.20
44C1	24	0.90 (0.84–0.97)	0.05	0.42 (0.27–0.63)^a^		0.18
44C1.1	17	0.93 (0.87–0.99)	0.03	0.39 (0.21–0.60)^a^		0.16
44C1.2	5	0.86 (0.75–0.97)	-0.01	0.05 (0.01–0.12)		0.01
44C1.3	2	0.93 (0.83–1.02)	0.02	0.04 (0.01–0.14)^a^		0.02
44C2	18	0.93 (0.90–0.97)	-0.02	0.40 (0.16–0.58)^a^		-0.05
44C2.1	6	0.86 (0.74–0.99)	-0.08	0.22 (0.01–0.51)		0.07
44C2.2	3	0.99 (0.99–1.00)	0.08	0.32 (0.00–0.62)		0.28
44C2.3	9	0.92 (0.88–0.96)	0.03	0.11 (0.04–0.21)^a^		0.00
44C3	5	0.98 (0.97–1.00)	0.07	0.29 (0.02–0.67)^a^		0.21
44C3.1	3	0.96 (0.90–1.03)	0.29	0.16 (0.00–0.50)		0.15
		**Weighted mean AUC**	**Δ**	**Weighted mean AUPR**		**Δ**
		0.93	+ 0.04	0.65		+ 0.08

Reported with the area under the receiver operating characteristic curve (AUC) and the area under the precision-recall curve (AUPR). The outcome measures for the most important groups. 95% confidence intervals (CI) are computed using bootstrapping. The “base case” corresponds to the Danis-Weber classes (AO/OTA 44A, 44B, and 44C). Outcomes with ≤ 1 instance are not reported. Radiographs at 400 × 400px resolution. ΔAUC and ΔAUPR was the difference in AUC and AUPR comparing the actively trained network to the pre-active training network at 256 × 256px resolution. Increasing resolution prior to active learning had no effect on performance﻿

^a^Indicates that the AUPR with 95% CI exceeds random AUPR

**Table 3:** Prediction outcomes for the external validation set (EVD)

FLINDERS – External validation dataset (EVD) (399 cases)					
**Malleolar fractures (44)**					
	**Cases**	**AUC (95% CI)**	**ΔAUC**	**AUPR (95% CI)**	**ΔAUPR**
Fracture	274	0.86 (0.82–0.89)	0.03	0.93 (0.91–0.95)^a^	0.00
**44A**					
Base	94	0.74 (0.68–0.80)	0.12	0.52 (0.40–0.61)^a^	0.20
44A1	93	0.75 (0.69–0.81)	0.14	0.57 (0.47–0.66)^a^	0.25
44A1.1	5	0.63 (0.33–0.94)	-0.07	0.04 (0.00–0.16)	0.02
44A1.2	28	0.78 (0.69–0.87)	0.15	0.26 (0.11–0.43)^a^	0.14
44A1.3	60	0.68 (0.61–0.76)	0.08	0.30 (0.20–0.41)^a^	0.10
**44B**					
Base	142	0.90 (0.87–0.93)	0.03	0.84 (0.78–0.89)^a^	0.03
44B1	116	0.84 (0.80–0.88)	0.03	0.68 (0.58–0.76)^a^	0.07
44B1.1	87	0.80 (0.75–0.85)	0.05	0.47 (0.37–0.56)^a^	0.06
44B1.2	27	0.80 (0.72–0.88)	0.02	0.19 (0.11–0.31)^a^	0.03
44B1.3	2	0.60 (0.17–1.02)	-0.30	0.01 (0.00–0.02)	-0.01
2	21	0.85 (0.75–0.94)	0.10	0.32 (0.17–0.50)^a^	0.19
44B2.1	18	0.85 (0.75–0.95)	0.12	0.33 (0.12–0.55)^a^	0.24
44B2.2	3	0.93 (0.88–0.99)	0.00	0.05 (0.00–0.17)	-0.03
44B3	5	0.82 (0.61–1.04)	-0.06	0.19 (0.01–0.58)	0.11
44B3.1	5	0.82 (0.63–1.02)	-0.05	0.12 (0.00–0.25)	0.07
**44C**					
Base	38	0.89 (0.82–0.96)	0.04	0.63 (0.46–0.78)^a^	-0.06
44C1	28	0.90 (0.84–0.96)	0.08	0.42 (0.26–0.65)^a^	0.07
44C1.1	27	0.90 (0.84–0.97)	0.07	0.44 (0.25–0.62)^a^	0.10
44C2	9	0.92 (0.82–1.01)	-0.04	0.19 (0.05–0.36)^a^	-0.40
44C2.1	9	0.90 (0.79–1.02)	-0.04	0.16 (0.05–0.31)^a^	-0.38
		**Weighted mean AUC**	**Δ**	**Weighted mean AUPR**	**Δ**
		0.83	+0.06	0.64	+0.07

Reported with the area under the receiver operating characteristic curve (AUC) and the area under the precision-recall curve (AUPR). The outcome measures for the most important groups. 95% confidence intervals (CI) are computed using bootstrapping. The “base case” corresponds to the Danis-Weber classes (AO/OTA 44A, 44B, and 44C). Outcomes with ≤ 1 instance are not reported. Radiographs at 400 × 400px resolution. ΔAUC and ΔAUPR was the difference in AUC and AUPR comparing the actively trained network to the pre-active training network at 256 × 256px resolution. Increasing resolution prior to active learning had no effect on performance﻿

^a^Indicates that the AUPR with 95% CI exceeds random AUPR

### Danderyd (IVD)

While the AUC was good for type A fractures in the IVD, AUPR was only better than chance for three outcomes (base case/ type “A”, subgroup A1.1 and group A3).

Type B fractures were the most numerous in the IVD. All had good to excellent AUC, and all had AUPR better than random, even rare outcomes such as B1.3, B2.3, and B3.3.

The network had excellent AUC and AUPR for type C fractures. The model performed better than random for the same type C outcomes in the Danderyd set (base, C1, C1.1, C2) as it did for the Flinders set, except C2.1.

The wAUC increased by 0.04 (from 0.89 to 0.93), and the wAUPR increased from 0.57 to 0.65 for the IVD. A random classifier would give a wAUPR of 0.23. See Table 2.

The model was less accurate for malleolar fracture detection ("base" AO/OTA 44) on the EVD than the IVD. The EVD dataset is less diverse, with fewer outcomes (23 vs. 36 AO/OTA outcomes). Notable was that fracture detection (fracture “yes”/”no”) had AUC 0.86 (0.82–0.89) for the EVD vs. AUC 0.95 (0.94–0.97) for the IVD.

### Flinders (EVD)

**Fig. 2:**
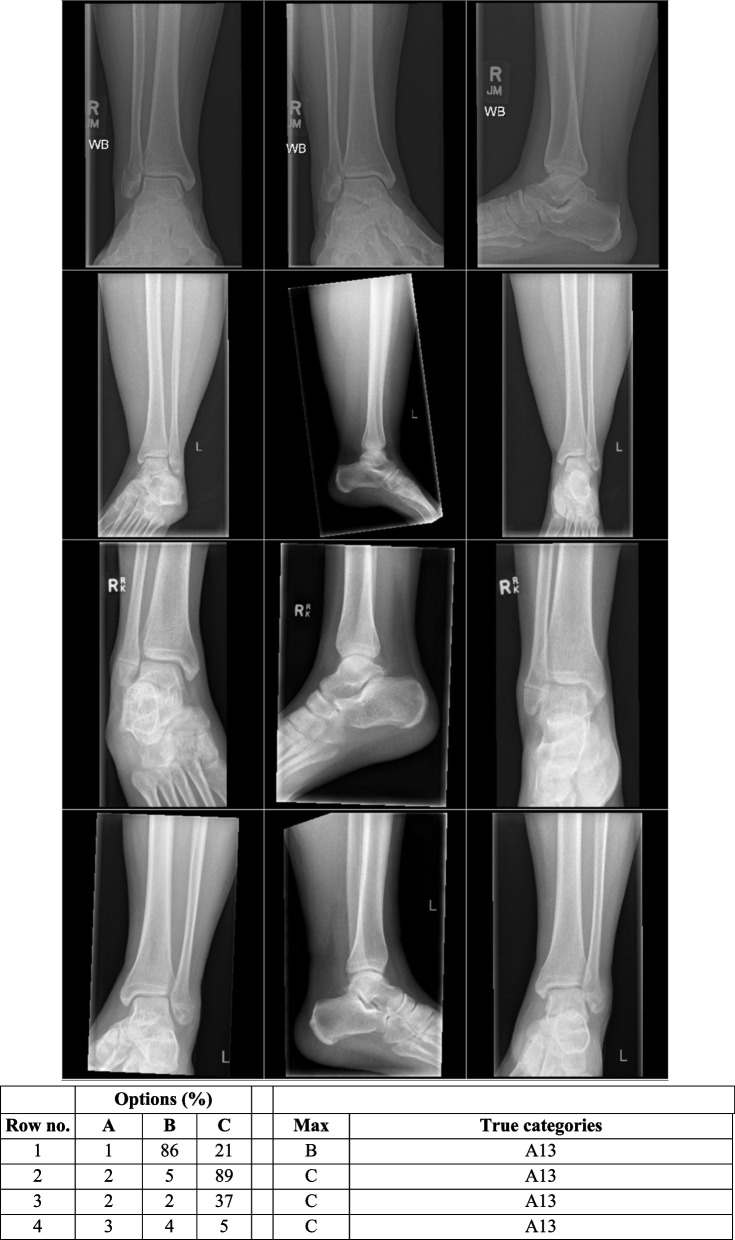
Incorrectly classified cases where the network failed to detect Type A, sorted from lowest probability to highest

Type A fractures were the second most numerous in the EVD, as was in group A1. Type A fractures had the lowest AUC, but only A1.1 performed worse than a random classifier measured in AUPR. However, there were few outcomes against which to measure performance. Figure 2 shows type A fractures and how the network classified them incorrectly.

For type B fractures, the base case performed well. While AUC was decent for all type B outcomes, four out of ten cases did not reach better AUPR than a random classifier (i.e., B1.3, B2.2, B3, and B3.1).

Type C fractures performed well, as did all four subclasses of type C outcomes. See Table 3.

**Fig. 3:**
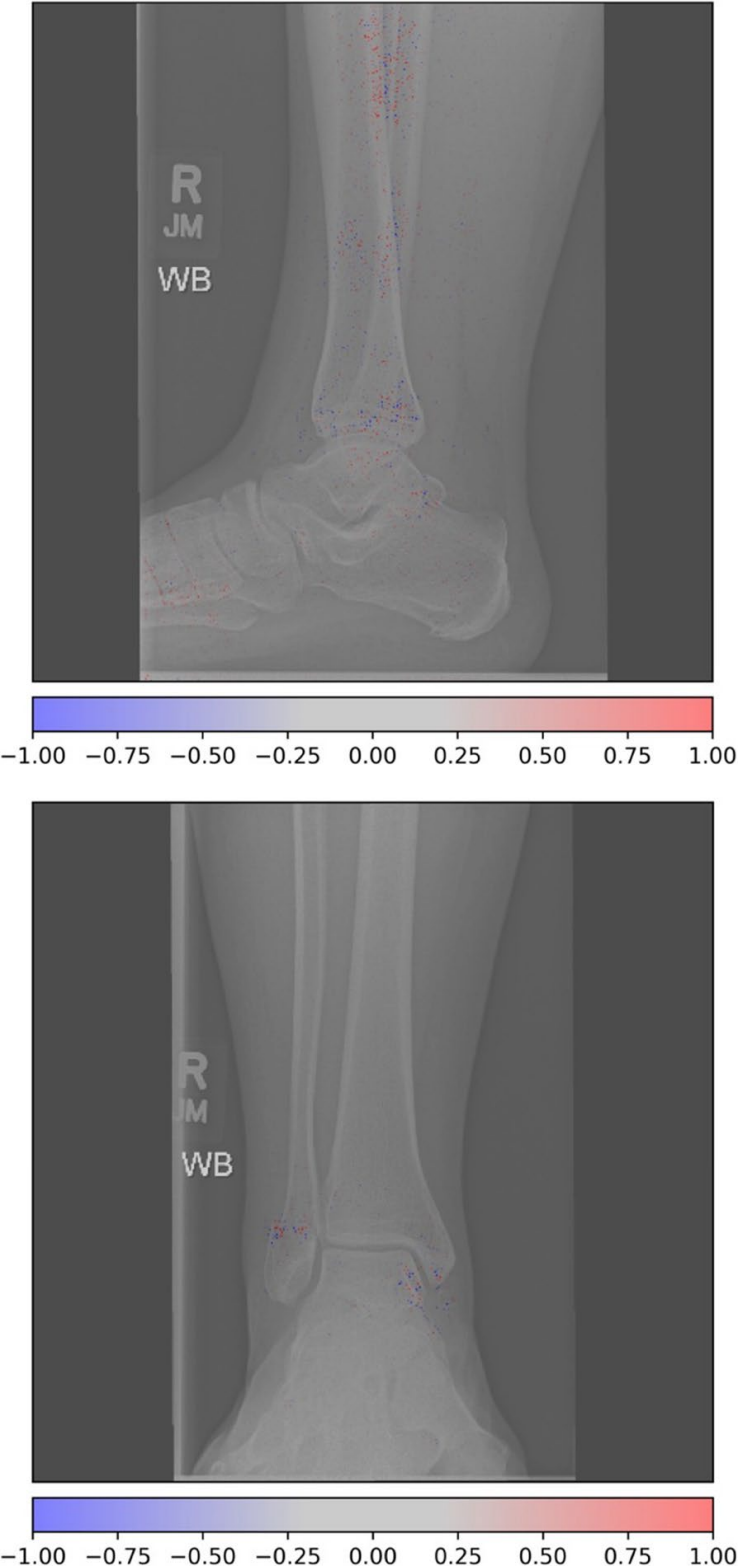
Activation heatmaps where a type 44A1.3 fracture is incorrectly classified as a type B fracture. The activations show what the model reacts to classify fractures. Study from the external validation data

**Fig. 4:**
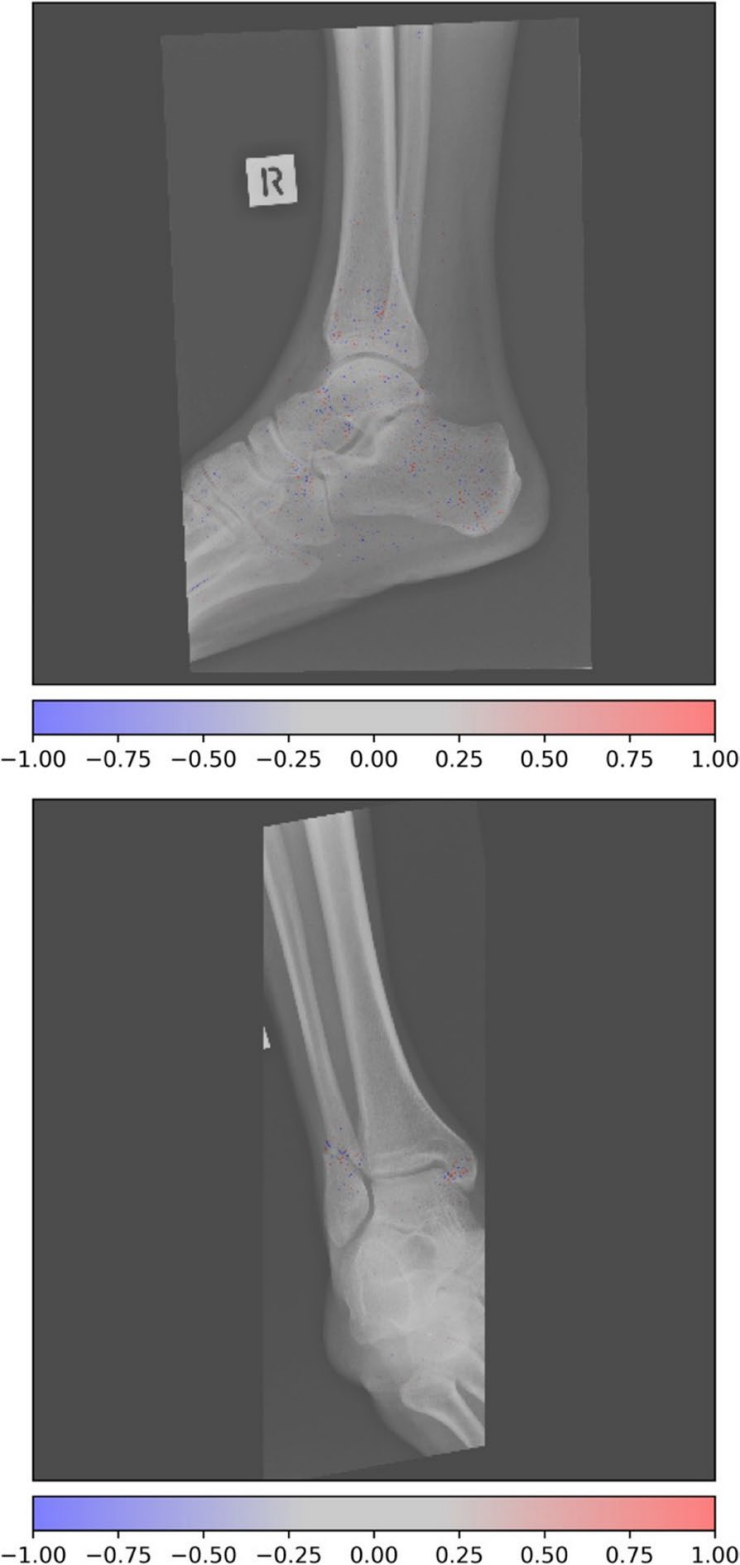
Activation heatmap of a type 44B1.2 fracture, incorrectly classified as type C fracture. The activations show what the model reacts to classify fractures. Study from the external validation data

Figure 3 shows an example of a type A1.3 fracture in the EVD, incorrectly classified as a type B fracture. Figure 4 shows examples of type B1.2 fracture incorrectly classified as type C.

Table 3 displays the change in performance for every class from active learning for the EVD. Most notable is a drop in performance for group C2 fractures, where group C2 and subgroup C2.1 decreased considerably in AUPR (-0.40 and -0.38, respectively). The wAUC increased by 0.06 (0.77 to 0.83), and the wAUPR increased from 0.57 to 0.63 for the EVD. A random classifier would give a wAUPR of 0.32.

#### Secondary outcomes: Intra-observer measurements (ICC and Cohen's Kappa)

Intra-observer measurements for malleolar fracture have ICC 0.86 and kappa 0.85, and for the type of fracture, 44A-C, ICC 0.76–0.84 and kappa 0.85–0.78. Less severe fractures, i.e., A1, B1, and C1, had higher kappa values than more severe cases, perhaps because they are more prevalent. ICC and kappa are poor for most other outcomes, and we consider the task challenging for humans. Several classes had no interobserver agreement (kappa 0), mainly because they were not represented in the EVD or were so few that any disagreement or agreement had a disproportionate influence. See Supplement 1, Table S2.

We report performance for secondary outcomes, like non-malleolar fractures, along with more in-depth data and complete experiment readouts in Supplement 3 (400 × 400 pixels) and Supplement 4 (256 × 256 pixels.). Supplement 4 reports the initial EVD performance before retraining and active learning.

## Discussion

This study aimed to externally validate a complex multi-label AO/OTA 2018 ankle fracture detection model. Few models are externally validated, and we found none as multifaceted as the AO/OTA in our study. In this study, we wanted to establish a baseline against which to compare future models. We found a gap in our model performance under external validation and reported a way of actively improving performance. We found that the model performed very well on an external validation set. Our model classified fractures much better than chance for all outcomes and indicates authentic learning utility for classifying ankle fractures in an external setting.

This study used the AO/OTA 2018 ankle classification system. A widely used alternative, alone or in conjunction, is the Lauge-Hansen (LH) system. The LH classification system aims to predict fracture patterns and ligamentous injuries based on injury mechanisms. Many studies have shown that LH is only partially valid or reproducible. Lindsjö, as far back as 1985, raised the question of poor reproducibility of LH between different populations based on previous studies [16]. Later studies repeated these findings of poor reproducibility [17–21]. An MRI study by Gardner et al. 2006 found that LH had limitations in predicting ligamentous injuries and soft-tissue damage [19]. These findings were replicated by Kwon et al. in 2010 and 2012 using actual injury footage [22–24]. Boszczyk et al. 2018, came to the same conclusion based on radiographs and patient-reported injury mechanisms [21]. Patton et al. 2022 came to similar conclusions based on CT and complete patient workups [25]. Both Michelson et al. and Haraguchi and Arminger failed to reproduce Lauge-Hansens's results in physical experiments. They concluded that the LH system could not be used to predict injury mechanisms or injury patterns [26, 27]. The AO/OTA standard launched the Danis-Weber system. Danis-Weber is based on the location of the lateral malleolus fracture about the syndesmosis. AO/OTA then extends the Danis-Weber classification to consider the medial and posterior malleolus injuries and grades fractures based on physical appearance [9, 28]. The main critique of the AO/OTA ankle system is that it is complex and that isolated medial malleolus fractures are treated as distal tibial fractures [20, 29].

Our goal was to develop AI models for rapid, easy, and accurate fracture classification and clinical decision-making. LH is not well suited to predicting injury mechanisms from radiographs in its current form, whereas AO/OTA is imaging-based. In the clinical context, AO/OTA (complete or simplified Danis-Weber) and LH are often used in conjunction to guide treatment decisions. The classifications are similar, and conversions between the two systems have been suggested, but no fully agreed-upon complete conversion exists [24, 30–34].

### Model training

The training of AI models often comes down to hidden factors and confounders that are only sometimes related to actual pathology detection. For example, in a multicenter study, Badgeley et al. found that logistic and healthcare system parameters were often responsible for prediction. Without them, performance fell to that of a random classifier [35]. Subjecting the model to another dataset exposes it to a different data distribution – called a dataset shift [36] – and is crucial for evaluating models. It should be integral to the model training and development stage. If the model only performs well on the data it was trained on or from one hospital, we can quantify this. It reduces the risk of presenting overfitted models as research progresses. In this study, the Flinders data has a different distribution and priori probabilities than the training data. For example, there were three times as many type A fractures. The Flinders data (e.g., EVD) had three images per study compared to four or more for Danderyd. The presence of follow-up images, e.g., weight-bearing one week after the trauma – was not a part of the network training. A non-displaced "weight-bearing" exam would signal a less severe injury to a human reviewer, whereas the network did not recognize this signal. We expected the IVD performance to be somewhat better. For both datasets, AUC and AUPR are better than random for all outcomes. Few AI models are validated, making it difficult to assess how general and transferable these models are to other settings and what performance we can expect in our study. For the three studies, Oliveira e Carmo et al. found performance was not affected dramatically for the validation set [3] (see Supplement 1, Table S1a.) However, those studies evaluated models with just two or three outcomes. The AO/OTA classification, as used in our research, had 40 outcomes for ankle fractures – not all mutually exclusive.

As we were dissatisfied with the performance of the EVD, we tried multiple strategies to improve performance. We increased image resolution, which did not affect EVD performance. We tried to drop views to make the training data resemble the EVD data (three standard views in EVD vs four or more in the training data). Neither had any performance effect, and we speculated that type A fracture signs had a too-discrete training signal for the network. Only after active training (i.e., additional training data focusing on the problematic type A classes) could we improve performance by increasing resolution. Yet, we did not see any rise in performance past 400 × 400px. However, it can be desirable to reverse this generalization process on the externally validated model in a clinical application, i.e., honing it in the local setting. This would be done by actively retraining the externally valid model on data from the clinic where it is being used.

To our knowledge, this is the first study that externally validates such a complex fracture classification model and raises the question of what we can expect. Our model performed well compared to other multinomial classifiers, even on EVD data [11, 37–43] (Supplement 1, Table S1b.) While we must take care when applying the algorithm to a new environment, it appears to work satisfactorily. Lim et al. (2014) found that many of the most common orthopedic procedures had poor evidence-based medicine support and were unnecessary [44]. We believe that tools such as this algorithm and evolutions could be part of the solution towards a more stringent and evidence-based treatment, for example, by reducing ambiguity in treatment decisions, identifying failure patterns, or automating data reporting to registries.

### Limitations and strengths

In alignment with our previous studies [9, 10], we initially tested our trained model on 256 × 256 radiographs but had difficulties capturing type A fractures. We attributed this to them being rarer in the training data. Type C injuries were also uncommon, but the network performed better. Our experience was that the radiological footprint of type A fractures was less clear as these injuries tended to be less severe. The model captured Type A fractures after increasing the radiograph resolution and actively training for them. We found no benefit in going beyond 400 × 400 radiographs for our data.

As not all outcomes were sufficiently prevalent in the test data, we could not quantify all outcomes with reasonable confidence intervals. This was evident in cases where upper AUC confidence interval bounds exceeded 1.00 (i.e., 100% accuracy). Similarly, outcomes with few test cases (5 or less) AUPR could not be shown to outperform random guessing.

We did not have the population distribution for either dataset. The original training data was anonymized upon collection and did not come with population parameters. It consisted of all available trauma radiographs at Danderyd at that time. We have only excluded pediatric ankles. This makes it representative of the area from where it was collected. The Flinders data concentrated on lateral malleolus injuries and excluded casts and displaced fractures but included weight-bearing images and exams that were not concurrent with the injury. Therefore, it was impossible to determine how representative the EVD was of training data regarding population.

CT and MRI scans and operative findings are essential to the AO/OTA classification. CT scans are considered the gold standard in visualization. Neither dataset had access to CT scans, MRI scans, or patient journals. The lack of additional modalities or patient records made ligamentous injuries more challenging to classify and can affect the ground truth. However, this accurately simulated the daily clinical practice in many clinical situations where the initial assessment is performed on a radiograph.

The validation sets were limited in size for a model with so many possible outcomes. Several outcomes were scarce in the EVD and IVD, making the variability extremely large.

We have only validated our model on this site. If we were to look at a different hospital, we would get different results. Of course, this is true with all external validation.

The software used in our previous study was unsuitable for this study's experiments. Due to the random nature of model training, the exact performance for the IVD and EVD will vary slightly. However, comparing the previous model with the updated software where active learning was performed would be erroneous. Instead, we replicate the initial experiment.

### Conclusions, interpretation & generalizability

To our knowledge, this is the first paper that externally validates a multi-label radiographic ankle fracture classifier of this complexity. Despite considerable differences in the data makeup, we illustrate active learning strategies to improve external validity. Our model could successfully be used for complex ankle fracture classification at a different hospital, which is not to say that it will work equally well at all hospitals. We concur with the literature that the clinical relevance of published AI models must be proved through external validation. As clinical AI goes beyond simply stating the obvious "fracture or no fracture," this becomes even more true.

## Supplementary Information

Supplementary Material 1.

Supplementary Material 2.

Supplementary Material 3.

Supplementary Material 4.

## Data Availability

Complete experiment outputs are available as supplements. Supplement 1 reports model training data, specifics, and parameters. Supplements 2 and 3 report experiment outputs for the models' post-active and pre-active learning. Due to patients' integrity risk, the AI model cannot be made openly available. However, it can be made available upon request for testing, review, or research purposes. The internal validation dataset will be made available through AIDA (https://medtech4health.se/aida/). The external validation set can be made available for review upon request to max.gordon@ki.se. JO and MG have used the Danderyd dataset (training and internal validation dataset) in other studies [9]. We also state that other studies have included the patient cohort, e.g., https://doi.org/10.1080/17453674.2017.1344459. To the primary author’s knowledge, the external validation data has not been used in another study.

## Data availability

Complete experiment outputs are available as supplements. Supplement 2 reports model training data, specifics, and parameters. Supplement 3 and 4 report experiment outputs for the models pre- and post active learning.

The AI model cannot be made openly available due to the integrity risk of patients. However, the model can be made available upon request for testing, review, or research purposes. The internal validation dataset will be made available through AIDA (https://medtech4health.se/aida/). The external validation set can be made available for review purposes upon request, to max.gordon@ki.se.

The Danderyd dataset (training and internal validation dataset) has been used in studies by JO and MG, e.g., https://doi.org/10.1080/17453674.2017.1344459. To the main author’s knowledge, the external validation data has not been used in another study.
