# Supplementary material for: External validation of an artificial intelligence multi-label deep learning model capable of ankle fracture classification
Source: BMC Musculoskelet Disord. 2024 Oct 4;25:788. doi: 10.1186/s12891-024-07884-2 (PMC11451058; doi:10.1186/s12891-024-07884-2)
Supplement: Supplementary file 1 — Supplementary Material 1. [file 12891_2024_7884_MOESM1_ESM.docx]

# Supplement 1

| Table S1. Related studies. The internal validation set (IVD) is from the original training data location. The external validation set (EVD) is data from different sites. Table 1a. compares external validation studies found by Oliveira e Carmo et al. 2021. Table 1b. compares studies that evaluate complex classifiers with many outcomes (multinomial classifiers) comparable to our study, where none is externally validated. ACC is accuracy. F1 is the F1-score. AUC is the area under the receiver-operator characteristic curve. |
| --- |
| \| **Study** \| **Anatomy** \| **Outcomes** \| **Exclusion** \| **Performance** \| \| --- \| --- \| --- \| --- \| --- \| \| **1a. External validation studies** \| \| \| \| \| \| Choi 2020 \| Elbow \| Supracondylar/no fracture \| Dislocation, not supracondylar fracture, bone dysplasia \| IVD: AUC 0.98  EVD: AUC 0.99 \| \| Blüthngen 2020 \| Wrists \| Intact/defect \| – \| IVD: AUC 0.93 EVD: AUC 0.80 \| \| Zhou 2020 \| Ribfractures CT-slices \| Old, healing, and fresh \| No fracture \| IVD: mean F1-score 0.84 EVD: F1-score 0.73 \| \| **1b. Complex classifiers** \| \| \| \| \| \| Dreizin 2021 \| Pelvic, CT-scans \| AO Type A-C No. outcomes: 3 \| Any operative treatment \| ACC 56-85% \| \| Lind 2021 \| Knee \| AO/OTA No. outcomes: 49 \| – \| AUC 0.87 for proximal tibia; 0.89 for patella; 0.89 distal femur \| \| Qi 2020 \| Femur \| AO/OTA No. outcomes: 11 \| Any disagreement between reviewers \| ACC 72% \| \| Tanzi 2020 \| Hip \| AO/OTA No. outcomes: 5 \| Type B and C \| AUC 86% \| \| Yoon 2020 \| Intertrochanteric CT 3D reconstructions \| AO/OTA type A No. outcomes: 10 \| No separation of patients between training and test \| ACC 97% and 90% \| \| Lee 2020 \| Femur \| AO/OTA A1-B3 No. outcomes: 9 \| Type C (too rare) \| AUC 0.87, F1-score 0.86, vs AUC 0.75, F1-score 0.5 depending on configuration \| \| Olsson 2021 \| Osteoarthritis \| Kellgren & Lawrence  No. outcomes: 5 \| – \| AUC 0.92 \| \| Chung 2018 \| Shoulder \| Neers’  No. outcomes: 5 \| Reviewer disagreement \| ACC 65-86%; AUC 0.90-0.98 \| |

| Table S2. Interclass correlation (ICC) between all four observers (two observers reviewed half of the test set each). Cohens’ Kappa measures intraobserver-reliability between reviewers MIJ and MG. 95% CI is computed using bootstrapping. |
| --- |
| \|  \| **ICC (95% CI)** \|  \|  \| **Kappa (95% CI)** \| \| --- \| --- \| --- \| --- \| --- \| \| **General** \|  \|  \| **General** \|  \| \| Fracture \|  \|  \| Fracture \|  \| \| Malleolar \| 0.86 (0.84 - 0.88) \|  \| Malleolar \| 0.85 (0.80 - 0.91) \| \| **Malleolar** \|  \|  \| **Malleolar** \|  \| \| **44A** \|  \|  \| **A** \|  \| \| base \| 0.84 (0.81 - 0.86) \|  \| base \| 0.85 (0.79 - 0.91) \| \| 44A1 \| 0.82 (0.79 - 0.84) \|  \| 1 \| 0.82 (0.76 - 0.89) \| \| 44A1.1 \| -0.02 (-0.07 - 0.04) \|  \| …1 \| -0.02 (-0.03 - -0.01) \| \| 44A1.2 \| 0.56 (0.51 - 0.61) \|  \| …2 \| 0.70 (0.55 - 0.84) \| \| 44A1.3 \| 0.71 (0.67 - 0.75) \|  \| …3 \| 0.81 (0.73 - 0.89) \| \| 44A2 \| 0.00 (-0.06 - 0.06) \|  \| 2 \| 0.00 (-0.01 - 0.00) \| \| 44A2.2 \| 0.00 (-0.06 - 0.06) \|  \| …2 \| 0.00 (-0.01 - 0.00) \| \| 44A2.3 \| 0.00 (-0.05 - 0.06) \|  \| …3 \| 0.00 (0.00 - 0.00) \| \| 44A3 \| 0.00 (-0.05 - 0.06) \|  \| 3 \| 1.0 (identical) \| \| 44A3.2 \| 0.00 (-0.05 - 0.06) \|  \| …2 \| 1.0 (identical) \| \| **44B** \|  \|  \| **B** \|  \| \| base \| 0.89 (0.87 - 0.91) \|  \| base \| 0.91 (0.87 - 0.95) \| \| 44B1 \| 0.76 (0.72 - 0.79) \|  \| 1 \| 0.84 (0.78 - 0.90) \| \| 44B1.1 \| 0.50 (0.44 - 0.55) \|  \| …1 \| 0.66 (0.57 - 0.75) \| \| 44B1.2 \| 0.24 (0.17 - 0.30) \|  \| …2 \| 0.46 (0.33 - 0.60) \| \| 44B1.3 \| 0.27 (0.20 - 0.33) \|  \| …3 \| 0.40 (-0.15 - 0.94) \| \| 44B2 \| 0.40 (0.34 - 0.46) \|  \| 2 \| 0.53 (0.32 - 0.74) \| \| 44B2.1 \| 0.37 (0.30 - 0.43) \|  \| …1 \| 0.39 (0.16 - 0.62) \| \| 44B2.2 \| 0.20 (0.14 - 0.26) \|  \| …2 \| 0.00 (0.00 - 0.00) \| \| 44B2.3 \| 0.00 (-0.06 - 0.06) \|  \| …3 \| 0.00 (0.00 - 0.00) \| \| 44B3 \| 0.12 (0.06 - 0.18) \|  \| 3 \| 0.00 (0.00 - 0.00) \| \| 44B3.1 \| 0.14 (0.08 - 0.20) \|  \| …1 \| 0.00 (0.00 - 0.00) \| \| 44B3.3 \| 0.00 (-0.05 - 0.06) \|  \| …3 \| 1.0 (identical) \| \| **44C** \|  \|  \| **C** \|  \| \| base \| 0.76 (0.72 - 0.79) \|  \| base \| 0.78 (0.67 - 0.90) \| \| 44C1 \| 0.63 (0.58 - 0.68) \|  \| 1 \| 0.67 (0.52 - 0.82) \| \| 44C1.1 \| 0.49 (0.43 - 0.55) \|  \| …1 \| 0.69 (0.54 - 0.84) \| \| 44C1.2 \| 0.00 (-0.06 - 0.06) \|  \| …2 \| 0.00 (0.00 - 0.00) \| \| 44C2 \| 0.47 (0.41 - 0.53) \|  \| 2 \| 0.49 (0.14 - 0.84) \| \| 44C2.1 \| 0.54 (0.49 - 0.60) \|  \| …1 \| 0.49 (0.14 - 0.84) \| \| 44C3 \| 0.00 (-0.06 - 0.06) \|  \| 3 \| 0.00 (0.00 - 0.00) \| \| 44C3.1 \| 0.00 (-0.05 - 0.06) \|  \| …1 \| 0.00 (0.00 - 0.00) \| \| 44C3.2 \| 0.00 (-0.05 - 0.06) \|  \| …2 \| 1.0 (identical) \| |
