# Supplementary material for: External validation of an artificial intelligence multi-label deep learning model capable of ankle fracture classification
Source: BMC Musculoskelet Disord. 2024 Oct 4;25:788. doi: 10.1186/s12891-024-07884-2 (PMC11451058; doi:10.1186/s12891-024-07884-2)
Supplement: Supplementary file 2 — Supplementary Material 2. [file 12891_2024_7884_MOESM2_ESM.html]

Ankle fracture resultat


# Ankle fracture resultat

- Participants
- Method of classification
  - Applied
    training
    - Teacher network
      generating pseudo-labels
- Base data
- Results
- Network
  results
  - Malleolar fracture classes
    - Average
      A-class
    - Average
      B-class
    - Average
      C-class
- Failure
  analysis
- Analysis of
  cases
  - Malleolar A
    - Correct
    - Failure
  - Malleolar B
    - Correct
    - Failure
  - Malleolar C
    - Correct
    - Failure
- Other fracture classes
  - Fibula without malleolar
    engagement
  - Tibia distal
  - Tibia
    diaphysis
  - Foot
    fractures
- Inter-rater results

Based on scenario **2x2x6x6\_all\_xray** from run
**20230303/15.35** after scene *I\_clean\_w\_dropout*
(without any resume).

# Participants

The training dataset consisted of 7,750 cases after removing 0 images
matching exclusion criteria. We separated 430 consisting of 400 unique
patients for validation during training while the remainder was used for
training. There was no patient overlap between the test and training
datasets, i.e. a patient can exist more than once but those cases will
be in either the training or the test set.

# Method of classification

Network used was a of a ResNet type, convolutional neural network,
that consisted in total of a 4-layer architecture with batch
normalization for each convolutional layer and adaptive max pool (see
Table 1 for structure). We randomly initialized the network and trained
using stochastic gradient descent. During training we alternated between
knee labels and other previously gathered fracture classification tasks
(48,826 exams) where each task shared the core network.

| Type | Blocks | Kernel Size | Filters | Group | no\_layers |
| --- | --- | --- | --- | --- | --- |
| ResNet block | 1 | 3x3 | 64 | Image | 2 |
| ResNet block | 1 | 3x3 | 64 | Image | 2 |
| ResNet block | 2 | 3x3 | 64 | Core | 4 |
| ResNet block | 2 | 3x3 | 128 | Core | 4 |
| ResNet block | 6 | 3x3 | 256 | Core | 12 |
| ResNet block | 6 | 3x3 | 512 | Core | 12 |
| Average for top  50% views | 1 | - | - | Pool | 0 |
| Convolutional | 1 | 1x1 | 72 | Classification | 1 |
| Fully connected | 1 | 1x1 | 4 | Classification | 1 |
| Fully connected | 1 | 1x1 | 4 | Classification | 1 |

The images were additionaly agumented with 2 jitters and separately
processed up until a max pool merged the features into per image or exam
depending on the type of outcome. In addition to the AO classification
outputs that were pooled at the per exam level we had image view
(i.e. AP, lateral, Oblique), medial clear space between talus and tibia,
and the distance between the tibia plafond and the talus as additional
outcomes.

## Applied training

```
## # A tibble: 1 × 6
##   name        epochs forward_noise learning_rate use_custom_dropout view_dropout
##   <chr>       <glue>         <int>         <dbl> <fct>                     <dbl>
## 1 I_clean_w_… 100 x…             0         0.025 No                         0.25
```

During all sessions we switched between the knee dataset and a
similarly labeled datasets that consisted of 48,826 exams. These were
also augmented with unlabeled images with the same proportion between
unlabeled as labeled in the knee dataset.

### Teacher network generating pseudo-labels

We used a parallel network, a teacher network, that had access to
both the image interpretation and the radiologist’s report in order to
generate pseudo labels. The training of this additional network was
performed in parallel with the the initialization and regularization
sessions where it got to learn the labels. During the final session we
extended the dataset with the unlabeled exams where we would use the
teacher’s psuedo labels when regular labels were lacking. The loss from
these pseudo labels was reduced to 10% in to avoid introducing too much
noise.

# Base data

|  | Train | | |  | Test | | |
| --- | --- | --- | --- | --- | --- | --- | --- |
|  | Yes | Maybe | No |  | Yes | Maybe | No |
| Distal tibia | | | | | | | |
| A | | | | | | | |
| base | 45 (0.6%) | 0 (0.0%) | 7705 (99.4%) |  | 14 (3.4%) | 0 (0.0%) | 395 (96.6%) |
| 1 | 27 (0.3%) | 0 (0.0%) | 7723 (99.7%) |  | 8 (2.0%) | 0 (0.0%) | 401 (98.0%) |
| 11 | 13 (0.2%) | 0 (0.0%) | 7737 (99.8%) |  | 8 (2.0%) | 0 (0.0%) | 401 (98.0%) |
| 12 | 6 (0.1%) | 0 (0.0%) | 7744 (99.9%) |  |  |  |  |
| 13 | 8 (0.1%) | 0 (0.0%) | 7742 (99.9%) |  |  |  |  |
| 2 | 11 (0.1%) | 0 (0.0%) | 7739 (99.9%) |  | 4 (1.0%) | 0 (0.0%) | 405 (99.0%) |
| 21 | 4 (0.1%) | 0 (0.0%) | 7746 (99.9%) |  | 1 (0.2%) | 0 (0.0%) | 408 (99.8%) |
| 22 | 4 (0.1%) | 0 (0.0%) | 7746 (99.9%) |  | 2 (0.5%) | 0 (0.0%) | 407 (99.5%) |
| 23 | 3 (0.0%) | 0 (0.0%) | 7747 (100.0%) |  | 1 (0.2%) | 0 (0.0%) | 408 (99.8%) |
| 3 | 7 (0.1%) | 0 (0.0%) | 7743 (99.9%) |  | 2 (0.5%) | 0 (0.0%) | 407 (99.5%) |
| 31 | 1 (0.0%) | 0 (0.0%) | 7749 (100.0%) |  |  |  |  |
| 32 | 3 (0.0%) | 0 (0.0%) | 7747 (100.0%) |  | 1 (0.2%) | 0 (0.0%) | 408 (99.8%) |
| 33 | 3 (0.0%) | 0 (0.0%) | 7747 (100.0%) |  | 1 (0.2%) | 0 (0.0%) | 408 (99.8%) |
| B | | | | | | | |
| base | 85 (1.1%) | 5 (0.1%) | 7660 (98.8%) |  | 6 (1.5%) | 2 (0.5%) | 401 (98.0%) |
| 1 | 78 (1.0%) | 5 (0.1%) | 7667 (98.9%) |  | 6 (1.5%) | 2 (0.5%) | 401 (98.0%) |
| 11 | 43 (0.6%) | 4 (0.1%) | 7703 (99.4%) |  | 4 (1.0%) | 2 (0.5%) | 403 (98.5%) |
| 12 | 34 (0.4%) | 1 (0.0%) | 7715 (99.5%) |  | 2 (0.5%) | 0 (0.0%) | 407 (99.5%) |
| 2 | 4 (0.1%) | 0 (0.0%) | 7746 (99.9%) |  |  |  |  |
| 21 | 1 (0.0%) | 0 (0.0%) | 7749 (100.0%) |  |  |  |  |
| 22 | 3 (0.0%) | 0 (0.0%) | 7747 (100.0%) |  |  |  |  |
| 3 | 1 (0.0%) | 0 (0.0%) | 7749 (100.0%) |  |  |  |  |
| 31 | 0 (0.0%) | 0 (0.0%) | 7750 (100.0%) |  |  |  |  |
| 32 | 1 (0.0%) | 0 (0.0%) | 7749 (100.0%) |  |  |  |  |
| C | | | | | | | |
| base | 21 (0.3%) | 0 (0.0%) | 7729 (99.7%) |  | 16 (3.9%) | 0 (0.0%) | 393 (96.1%) |
| 1 | 9 (0.1%) | 0 (0.0%) | 7741 (99.9%) |  | 4 (1.0%) | 0 (0.0%) | 405 (99.0%) |
| 11 | 2 (0.0%) | 0 (0.0%) | 7748 (100.0%) |  | 1 (0.2%) | 0 (0.0%) | 408 (99.8%) |
| 13 | 7 (0.1%) | 0 (0.0%) | 7743 (99.9%) |  | 3 (0.7%) | 0 (0.0%) | 406 (99.3%) |
| 2 | 3 (0.0%) | 0 (0.0%) | 7747 (100.0%) |  | 5 (1.2%) | 0 (0.0%) | 404 (98.8%) |
| 22 | 2 (0.0%) | 0 (0.0%) | 7748 (100.0%) |  | 1 (0.2%) | 0 (0.0%) | 408 (99.8%) |
| 23 | 1 (0.0%) | 0 (0.0%) | 7749 (100.0%) |  | 4 (1.0%) | 0 (0.0%) | 405 (99.0%) |
| 3 | 9 (0.1%) | 0 (0.0%) | 7741 (99.9%) |  | 7 (1.7%) | 0 (0.0%) | 402 (98.3%) |
| 31 | 3 (0.0%) | 0 (0.0%) | 7747 (100.0%) |  | 1 (0.2%) | 0 (0.0%) | 408 (99.8%) |
| 32 | 1 (0.0%) | 0 (0.0%) | 7749 (100.0%) |  | 3 (0.7%) | 0 (0.0%) | 406 (99.3%) |
| 33 | 5 (0.1%) | 0 (0.0%) | 7745 (99.9%) |  | 3 (0.7%) | 0 (0.0%) | 406 (99.3%) |
| Fibula | | | | | | | |
| Modifier | 74 (1.0%) | 2 (0.0%) | 2690 (34.7%) |  |  |  |  |
| Modifier 1 | 29 (0.4%) | 2 (0.0%) | 2735 (35.3%) |  |  |  |  |
| Modifier 13 | 8 (0.1%) | 0 (0.0%) | 2758 (35.6%) |  |  |  |  |
| Modifier 14 | 4 (0.1%) | 0 (0.0%) | 2762 (35.6%) |  |  |  |  |
| Modifier 2 | 37 (0.5%) | 0 (0.0%) | 2729 (35.2%) |  |  |  |  |
| Modifier 3 | 7 (0.1%) | 0 (0.0%) | 2759 (35.6%) |  |  |  |  |
| Modifier 3 b | 7 (0.1%) | 0 (0.0%) | 2759 (35.6%) |  |  |  |  |
| Modifier 4 | 63 (0.8%) | 2 (0.0%) | 2701 (34.9%) |  |  |  |  |
| Modifier 7 | 3 (0.0%) | 0 (0.0%) | 2763 (35.7%) |  |  |  |  |
| Modifier 9 | 2 (0.0%) | 0 (0.0%) | 2764 (35.7%) |  |  |  |  |
| 1 | | | | | | | |
| base | 0 (0.0%) | 0 (0.0%) | 7750 (100.0%) |  |  |  |  |
| A | 0 (0.0%) | 0 (0.0%) | 7750 (100.0%) |  |  |  |  |
| 2 | | | | | | | |
| base | 82 (1.1%) | 2 (0.0%) | 7666 (98.9%) |  | 5 (1.2%) | 0 (0.0%) | 404 (98.8%) |
| A | 48 (0.6%) | 2 (0.0%) | 7700 (99.4%) |  | 3 (0.7%) | 0 (0.0%) | 406 (99.3%) |
| B | 34 (0.4%) | 0 (0.0%) | 7716 (99.6%) |  | 2 (0.5%) | 0 (0.0%) | 407 (99.5%) |
| …→a | 1 (0.0%) | 0 (0.0%) | 2765 (35.7%) |  |  |  |  |
| …→b | 16 (0.2%) | 0 (0.0%) | 2750 (35.5%) |  |  |  |  |
| …→c | 23 (0.3%) | 1 (0.0%) | 2911 (37.6%) |  |  |  |  |
| 3 | | | | | | | |
| base | 110 (1.4%) | 1 (0.0%) | 7639 (98.6%) |  | 32 (7.8%) | 0 (0.0%) | 377 (92.2%) |
| A | 86 (1.1%) | 1 (0.0%) | 7663 (98.9%) |  | 16 (3.9%) | 0 (0.0%) | 393 (96.1%) |
| B | 24 (0.3%) | 0 (0.0%) | 7726 (99.7%) |  | 16 (3.9%) | 0 (0.0%) | 393 (96.1%) |
| General | | | | | | | |
| Degenerative | 133 (1.7%) | 0 (0.0%) | 2633 (34.0%) |  |  |  |  |
| Dislocation | 74 (1.0%) | 0 (0.0%) | 2692 (34.7%) |  |  |  |  |
| Dislocation lateral | 60 (0.8%) | 0 (0.0%) | 2706 (34.9%) |  |  |  |  |
| Dislocation posterior | 10 (0.1%) | 0 (0.0%) | 2756 (35.6%) |  |  |  |  |
| Dislocation repositioned | 14 (0.2%) | 0 (0.0%) | 2752 (35.5%) |  |  |  |  |
| Implants | 145 (1.9%) | 0 (0.0%) | 2621 (33.8%) |  |  |  |  |
| Previous | 148 (1.9%) | 0 (0.0%) | 2787 (36.0%) |  |  |  |  |
| Previous early | 15 (0.2%) | 0 (0.0%) | 2751 (35.5%) |  |  |  |  |
| Previous healed | 104 (1.3%) | 0 (0.0%) | 2662 (34.3%) |  |  |  |  |
| Previous late | 22 (0.3%) | 0 (0.0%) | 2744 (35.4%) |  |  |  |  |
| Fracture | | | | | | | |
| base | 3624 (46.8%) | 117 (1.5%) | 4009 (51.7%) |  | 304 (74.3%) | 12 (2.9%) | 93 (22.7%) |
| Fibula | 201 (2.6%) | 5 (0.1%) | 7544 (97.3%) |  | 37 (9.0%) | 0 (0.0%) | 372 (91.0%) |
| Foot | 359 (4.6%) | 54 (0.7%) | 7337 (94.7%) |  | 35 (8.6%) | 5 (1.2%) | 369 (90.2%) |
| Lower leg | 3312 (42.7%) | 116 (1.5%) | 4322 (55.8%) |  | 273 (66.7%) | 7 (1.7%) | 129 (31.5%) |
| Malleolar | 2836 (36.6%) | 99 (1.3%) | 4815 (62.1%) |  | 211 (51.6%) | 5 (1.2%) | 193 (47.2%) |
| Tibia | 420 (5.4%) | 13 (0.2%) | 7317 (94.4%) |  | 63 (15.4%) | 2 (0.5%) | 344 (84.1%) |
| Tibia diaphyseal | 169 (2.2%) | 1 (0.0%) | 7580 (97.8%) |  | 27 (6.6%) | 0 (0.0%) | 382 (93.4%) |
| Tibia distal | 246 (3.2%) | 7 (0.1%) | 7497 (96.7%) |  | 36 (8.8%) | 0 (0.0%) | 373 (91.2%) |
| Malleolar | | | | | | | |
| Modifier | 1013 (13.1%) | 28 (0.4%) | 1725 (22.3%) |  |  |  |  |
| Modifier 1 | 615 (7.9%) | 21 (0.3%) | 2130 (27.5%) |  |  |  |  |
| Modifier 13 | 9 (0.1%) | 0 (0.0%) | 2757 (35.6%) |  |  |  |  |
| Modifier 2 | 215 (2.8%) | 1 (0.0%) | 2550 (32.9%) |  |  |  |  |
| Modifier 3 | 20 (0.3%) | 0 (0.0%) | 2746 (35.4%) |  |  |  |  |
| Modifier 3 a | 13 (0.2%) | 0 (0.0%) | 2753 (35.5%) |  |  |  |  |
| Modifier 3 b | 6 (0.1%) | 0 (0.0%) | 2760 (35.6%) |  |  |  |  |
| Modifier 4 | 974 (12.6%) | 28 (0.4%) | 1764 (22.8%) |  |  |  |  |
| Modifier 5 | 49 (0.6%) | 0 (0.0%) | 2717 (35.1%) |  |  |  |  |
| Modifier 5 b | 4 (0.1%) | 0 (0.0%) | 2762 (35.6%) |  |  |  |  |
| Modifier 5 c | 2 (0.0%) | 0 (0.0%) | 2764 (35.7%) |  |  |  |  |
| Modifier 5 d | 39 (0.5%) | 0 (0.0%) | 2727 (35.2%) |  |  |  |  |
| Modifier 5 f | 4 (0.1%) | 0 (0.0%) | 2762 (35.6%) |  |  |  |  |
| Modifier 6 | 21 (0.3%) | 0 (0.0%) | 2745 (35.4%) |  |  |  |  |
| Modifier 6 d | 21 (0.3%) | 0 (0.0%) | 2745 (35.4%) |  |  |  |  |
| Modifier 7 | 19 (0.2%) | 0 (0.0%) | 2747 (35.4%) |  |  |  |  |
| Modifier 9 | 14 (0.2%) | 0 (0.0%) | 2752 (35.5%) |  |  |  |  |
| A | | | | | | | |
| base | 881 (11.4%) | 86 (1.1%) | 6783 (87.5%) |  | 27 (6.6%) | 5 (1.2%) | 377 (92.2%) |
| 1 | 687 (8.9%) | 79 (1.0%) | 6984 (90.1%) |  | 18 (4.4%) | 4 (1.0%) | 387 (94.6%) |
| 11 | 144 (1.9%) | 50 (0.6%) | 7556 (97.5%) |  | 4 (1.0%) | 2 (0.5%) | 403 (98.5%) |
| 12 | 301 (3.9%) | 13 (0.2%) | 7436 (95.9%) |  | 6 (1.5%) | 1 (0.2%) | 402 (98.3%) |
| 13 | 240 (3.1%) | 16 (0.2%) | 7494 (96.7%) |  | 8 (2.0%) | 1 (0.2%) | 400 (97.8%) |
| 2 | 173 (2.2%) | 6 (0.1%) | 7571 (97.7%) |  | 7 (1.7%) | 0 (0.0%) | 402 (98.3%) |
| 21 | 141 (1.8%) | 6 (0.1%) | 7603 (98.1%) |  | 5 (1.2%) | 0 (0.0%) | 404 (98.8%) |
| 22 | 6 (0.1%) | 0 (0.0%) | 7744 (99.9%) |  |  |  |  |
| 23 | 26 (0.3%) | 0 (0.0%) | 7724 (99.7%) |  | 2 (0.5%) | 0 (0.0%) | 407 (99.5%) |
| 3 | 20 (0.3%) | 0 (0.0%) | 7730 (99.7%) |  | 2 (0.5%) | 0 (0.0%) | 407 (99.5%) |
| 31 | 14 (0.2%) | 0 (0.0%) | 7736 (99.8%) |  |  |  |  |
| 33 | 6 (0.1%) | 0 (0.0%) | 7744 (99.9%) |  | 2 (0.5%) | 0 (0.0%) | 407 (99.5%) |
| B | | | | | | | |
| base | 1601 (20.7%) | 9 (0.1%) | 6140 (79.2%) |  | 137 (33.5%) | 0 (0.0%) | 272 (66.5%) |
| 1 | 865 (11.2%) | 8 (0.1%) | 6877 (88.7%) |  | 67 (16.4%) | 0 (0.0%) | 342 (83.6%) |
| 11 | 635 (8.2%) | 8 (0.1%) | 7107 (91.7%) |  | 39 (9.5%) | 0 (0.0%) | 370 (90.5%) |
| 12 | 217 (2.8%) | 0 (0.0%) | 7533 (97.2%) |  | 26 (6.4%) | 0 (0.0%) | 383 (93.6%) |
| 13 | 12 (0.2%) | 0 (0.0%) | 7738 (99.8%) |  | 2 (0.5%) | 0 (0.0%) | 407 (99.5%) |
| 1→o | 5 (0.1%) | 0 (0.0%) | 2761 (35.6%) |  |  |  |  |
| 1→u | 5 (0.1%) | 0 (0.0%) | 2761 (35.6%) |  |  |  |  |
| 2 | 350 (4.5%) | 0 (0.0%) | 7400 (95.5%) |  | 38 (9.3%) | 0 (0.0%) | 371 (90.7%) |
| 21 | 181 (2.3%) | 0 (0.0%) | 7569 (97.7%) |  | 20 (4.9%) | 0 (0.0%) | 389 (95.1%) |
| 22 | 148 (1.9%) | 0 (0.0%) | 7602 (98.1%) |  | 16 (3.9%) | 0 (0.0%) | 393 (96.1%) |
| 23 | 21 (0.3%) | 0 (0.0%) | 7729 (99.7%) |  | 2 (0.5%) | 0 (0.0%) | 407 (99.5%) |
| 23r | 2 (0.0%) | 0 (0.0%) | 2764 (35.7%) |  |  |  |  |
| 23u | 3 (0.0%) | 0 (0.0%) | 2763 (35.7%) |  |  |  |  |
| 2→o | 4 (0.1%) | 0 (0.0%) | 2762 (35.6%) |  |  |  |  |
| 2→u | 49 (0.6%) | 0 (0.0%) | 2717 (35.1%) |  |  |  |  |
| 3 | 384 (5.0%) | 1 (0.0%) | 7365 (95.0%) |  | 32 (7.8%) | 0 (0.0%) | 377 (92.2%) |
| 31 | 119 (1.5%) | 0 (0.0%) | 7631 (98.5%) |  | 12 (2.9%) | 0 (0.0%) | 397 (97.1%) |
| 32 | 213 (2.7%) | 1 (0.0%) | 7536 (97.2%) |  | 13 (3.2%) | 0 (0.0%) | 396 (96.8%) |
| 33 | 52 (0.7%) | 0 (0.0%) | 7698 (99.3%) |  | 6 (1.5%) | 0 (0.0%) | 403 (98.5%) |
| 3→o | 1 (0.0%) | 0 (0.0%) | 2765 (35.7%) |  |  |  |  |
| 3→u | 28 (0.4%) | 0 (0.0%) | 2738 (35.3%) |  |  |  |  |
| C | | | | | | | |
| base | 351 (4.5%) | 3 (0.0%) | 7396 (95.4%) |  | 47 (11.5%) | 0 (0.0%) | 362 (88.5%) |
| 1 | 182 (2.3%) | 0 (0.0%) | 7568 (97.7%) |  | 24 (5.9%) | 0 (0.0%) | 385 (94.1%) |
| 11 | 131 (1.7%) | 0 (0.0%) | 7619 (98.3%) |  | 17 (4.2%) | 0 (0.0%) | 392 (95.8%) |
| 12 | 24 (0.3%) | 0 (0.0%) | 7726 (99.7%) |  | 5 (1.2%) | 0 (0.0%) | 404 (98.8%) |
| 13 | 27 (0.3%) | 0 (0.0%) | 7723 (99.7%) |  | 2 (0.5%) | 0 (0.0%) | 407 (99.5%) |
| 2 | 107 (1.4%) | 0 (0.0%) | 7643 (98.6%) |  | 18 (4.4%) | 0 (0.0%) | 391 (95.6%) |
| 21 | 39 (0.5%) | 0 (0.0%) | 7711 (99.5%) |  | 6 (1.5%) | 0 (0.0%) | 403 (98.5%) |
| 22 | 24 (0.3%) | 0 (0.0%) | 7726 (99.7%) |  | 3 (0.7%) | 0 (0.0%) | 406 (99.3%) |
| 23 | 44 (0.6%) | 0 (0.0%) | 7706 (99.4%) |  | 9 (2.2%) | 0 (0.0%) | 400 (97.8%) |
| 3 | 62 (0.8%) | 3 (0.0%) | 7685 (99.2%) |  | 5 (1.2%) | 0 (0.0%) | 404 (98.8%) |
| 31 | 16 (0.2%) | 1 (0.0%) | 7733 (99.8%) |  | 3 (0.7%) | 0 (0.0%) | 406 (99.3%) |
| 32 | 9 (0.1%) | 0 (0.0%) | 7741 (99.9%) |  | 1 (0.2%) | 0 (0.0%) | 408 (99.8%) |
| 33 | 37 (0.5%) | 2 (0.0%) | 7711 (99.5%) |  | 1 (0.2%) | 0 (0.0%) | 408 (99.8%) |
| 3→p | 6 (0.1%) | 0 (0.0%) | 2760 (35.6%) |  |  |  |  |
| 3→r | 13 (0.2%) | 1 (0.0%) | 2752 (35.5%) |  |  |  |  |
| 3→s | 7 (0.1%) | 0 (0.0%) | 2759 (35.6%) |  |  |  |  |
| …→→t | 23 (0.3%) | 0 (0.0%) | 2743 (35.4%) |  |  |  |  |
| …→→u | 13 (0.2%) | 0 (0.0%) | 2753 (35.5%) |  |  |  |  |
| Tibia, diaphyseal | | | | | | | |
| A | | | | | | | |
| base | 53 (0.7%) | 0 (0.0%) | 7697 (99.3%) |  | 16 (3.9%) | 0 (0.0%) | 393 (96.1%) |
| 1 | 42 (0.5%) | 0 (0.0%) | 7708 (99.5%) |  | 16 (3.9%) | 0 (0.0%) | 393 (96.1%) |
| 2 | 8 (0.1%) | 0 (0.0%) | 7742 (99.9%) |  |  |  |  |
| 3 | 3 (0.0%) | 0 (0.0%) | 7747 (100.0%) |  |  |  |  |
| B | | | | | | | |
| base | 26 (0.3%) | 0 (0.0%) | 7724 (99.7%) |  | 7 (1.7%) | 0 (0.0%) | 402 (98.3%) |
| 2 | 24 (0.3%) | 0 (0.0%) | 7726 (99.7%) |  | 7 (1.7%) | 0 (0.0%) | 402 (98.3%) |
| 3 | 2 (0.0%) | 0 (0.0%) | 7748 (100.0%) |  | 0 (0.0%) | 0 (0.0%) | 409 (100.0%) |
| C | | | | | | | |
| base | 1 (0.0%) | 0 (0.0%) | 7749 (100.0%) |  | 4 (1.0%) | 0 (0.0%) | 405 (99.0%) |
| 2 | 1 (0.0%) | 0 (0.0%) | 7749 (100.0%) |  | 3 (0.7%) | 0 (0.0%) | 406 (99.3%) |
| 3 |  |  |  |  | 1 (0.2%) | 0 (0.0%) | 408 (99.8%) |

# Results

A total of 8,205 examinations were used in the experiment. 7,366
examinations were used for training and validation and 409 examinations
(400 unique patients) were withheld into the test set, with no patient
overlap.

# Network results

## Malleolar fracture classes

Results compared for the images prepared by the specialist

|  |  |  |  |  |  |  |  |
| --- | --- | --- | --- | --- | --- | --- | --- |
| The outcome measures for the most important groups. Criterion based on Youden’s Index (Youden 1950; Aoki et al. 1997; Shapiro 1999; Greiner et al. 2000) defined as YI(c)=max\_{c}(Se(c)+Sp(c)-1). This is identical (from an optimization point of view) to the method that maximizes the sum of Sensitivity and Specificity (Albert and Harris 1987; Zweig and Campbell 1993) and to the criterion that maximizes concordance, wich is a monotone function of the AUC | | | | | | | |
|  |  |  | Measurements | | | | |
|  | Cases (n=409) |  | Sensitivity (%) | Specificity (%) | Youden’s J | AUC (95% CI) | prAUC (95% CI) |
| Base | 216 |  | 91 | 92 | 0.83 | 0.96 (0.94 to 0.98) | 0.96 (0.94 to 0.98) |
| A | | | | | | | |
| Base | 32 |  | 63 | 93 | 0.56 | 0.86 (0.79 to 0.93) | 0.42 (0.19 to 0.59) |
| 1 | 22 |  | 83 | 70 | 0.53 | 0.86 (0.77 to 0.94) | 0.28 (0.11 to 0.47) |
| 11 | 6 |  | 100 | 77 | 0.77 | 0.90 (0.78 to 1.02) | 0.27 (0.00 to 0.51) |
| 12 | 7 |  | 83 | 80 | 0.63 | 0.86 (0.68 to 1.04) | 0.20 (0.00 to 0.49) |
| 13 | 9 |  | 100 | 51 | 0.51 | 0.79 (0.66 to 0.93) | 0.10 (0.01 to 0.26) |
| 2 | 7 |  | 100 | 93 | 0.93 | 0.98 (0.96 to 1.00) | 0.48 (0.09 to 0.74) |
| 21 | 5 |  | 100 | 93 | 0.93 | 0.98 (0.95 to 1.01) | 0.39 (0.03 to 0.63) |
| 23 | 2 |  | 100 | 98 | 0.98 | 0.99 (0.97 to 1.01) | 0.20 (0.00 to 0.39) |
| 3 | 2 |  | 100 | 97 | 0.97 | 0.98 (0.96 to 1.00) | 0.12 (0.03 to 0.36) |
| B | | | | | | | |
| Base | 137 |  | 92 | 89 | 0.81 | 0.95 (0.93 to 0.98) | 0.91 (0.86 to 0.94) |
| 1 | 67 |  | 97 | 85 | 0.82 | 0.95 (0.93 to 0.97) | 0.73 (0.61 to 0.82) |
| 11 | 39 |  | 97 | 79 | 0.76 | 0.90 (0.87 to 0.93) | 0.37 (0.25 to 0.51) |
| 12 | 26 |  | 92 | 84 | 0.77 | 0.92 (0.88 to 0.95) | 0.32 (0.18 to 0.50) |
| 13 | 2 |  | 100 | 90 | 0.90 | 0.91 (0.87 to 0.95) | 0.02 (0.01 to 0.07) |
| 2 | 38 |  | 82 | 78 | 0.60 | 0.85 (0.79 to 0.91) | 0.39 (0.25 to 0.55) |
| 21 | 20 |  | 90 | 77 | 0.67 | 0.90 (0.85 to 0.96) | 0.38 (0.16 to 0.58) |
| 22 | 16 |  | 81 | 90 | 0.71 | 0.89 (0.81 to 0.98) | 0.30 (0.13 to 0.50) |
| 23 | 2 |  | 100 | 69 | 0.69 | 0.83 (0.55 to 1.11) | 0.03 (0.00 to 0.12) |
| 3 | 32 |  | 100 | 72 | 0.72 | 0.92 (0.88 to 0.95) | 0.47 (0.28 to 0.62) |
| 31 | 12 |  | 92 | 82 | 0.74 | 0.91 (0.87 to 0.96) | 0.16 (0.07 to 0.27) |
| 32 | 13 |  | 100 | 75 | 0.75 | 0.91 (0.86 to 0.95) | 0.18 (0.07 to 0.36) |
| 33 | 6 |  | 100 | 97 | 0.97 | 0.98 (0.97 to 0.99) | 0.26 (0.07 to 0.50) |
| C | | | | | | | |
| Base | 47 |  | 94 | 82 | 0.75 | 0.94 (0.91 to 0.97) | 0.73 (0.56 to 0.84) |
| 1 | 24 |  | 79 | 90 | 0.69 | 0.91 (0.86 to 0.96) | 0.37 (0.19 to 0.57) |
| 11 | 17 |  | 94 | 88 | 0.82 | 0.94 (0.90 to 0.99) | 0.37 (0.19 to 0.58) |
| 12 | 5 |  | 100 | 66 | 0.66 | 0.82 (0.73 to 0.90) | 0.03 (0.01 to 0.06) |
| 13 | 2 |  | 100 | 85 | 0.85 | 0.91 (0.79 to 1.03) | 0.03 (0.01 to 0.14) |
| 2 | 18 |  | 100 | 85 | 0.85 | 0.95 (0.92 to 0.97) | 0.46 (0.19 to 0.63) |
| 21 | 6 |  | 100 | 73 | 0.73 | 0.89 (0.80 to 0.99) | 0.24 (0.01 to 0.51) |
| 22 | 3 |  | 100 | 98 | 0.98 | 0.99 (0.97 to 1.00) | 0.29 (0.00 to 0.57) |
| 23 | 9 |  | 100 | 85 | 0.85 | 0.92 (0.88 to 0.96) | 0.12 (0.04 to 0.22) |
| 3 | 5 |  | 100 | 96 | 0.96 | 0.98 (0.97 to 1.00) | 0.26 (0.03 to 0.53) |
| 31 | 3 |  | 100 | 92 | 0.92 | 0.97 (0.92 to 1.02) | 0.22 (0.00 to 0.67) |

|  |  |
| --- | --- |
| Mean weighted summary statistics where each column is multiplied by the number of cases and then divided by the total. This is applied to for cases matching ‘^(ao\_malleolar\_|general\_Malleolar)’, total of 35 measurements. | |
| Statisic | Mean |
| Sensitivity | 0.91 |
| Specificity | 0.86 |
| YoudenJ | 0.77 |
| AUC | 0.93 |
| prAUC | 0.64 |

### Average A-class

|  |  |
| --- | --- |
| Mean weighted summary statistics where each column is multiplied by the number of cases and then divided by the total. This is applied to for cases matching ‘^(ao\_malleolar\_A)’, total of 9 measurements. | |
| Statisic | Mean |
| Sensitivity | 0.82 |
| Specificity | 0.81 |
| YoudenJ | 0.63 |
| AUC | 0.88 |
| prAUC | 0.32 |

### Average B-class

|  |  |
| --- | --- |
| Mean weighted summary statistics where each column is multiplied by the number of cases and then divided by the total. This is applied to for cases matching ‘^(ao\_malleolar\_B)’, total of 13 measurements. | |
| Statisic | Mean |
| Sensitivity | 0.93 |
| Specificity | 0.83 |
| YoudenJ | 0.76 |
| AUC | 0.93 |
| prAUC | 0.60 |

### Average C-class

|  |  |
| --- | --- |
| Mean weighted summary statistics where each column is multiplied by the number of cases and then divided by the total. This is applied to for cases matching ‘^(ao\_malleolar\_C)’, total of 12 measurements. | |
| Statisic | Mean |
| Sensitivity | 0.94 |
| Specificity | 0.85 |
| YoudenJ | 0.78 |
| AUC | 0.93 |
| prAUC | 0.45 |

# Failure analysis

# Analysis of cases

## Malleolar A

### Correct

### Failure

## Malleolar B

### Correct

### Failure

## Malleolar C

### Correct

### Failure

# Other fracture classes

## Fibula without malleolar engagement

|  |  |  |  |  |  |  |  |
| --- | --- | --- | --- | --- | --- | --- | --- |
| The outcome measures for the most important groups. Criterion based on Youden’s Index (Youden 1950; Aoki et al. 1997; Shapiro 1999; Greiner et al. 2000) defined as YI(c)=max\_{c}(Se(c)+Sp(c)-1). This is identical (from an optimization point of view) to the method that maximizes the sum of Sensitivity and Specificity (Albert and Harris 1987; Zweig and Campbell 1993) and to the criterion that maximizes concordance, wich is a monotone function of the AUC | | | | | | | |
|  |  |  | Measurements | | | | |
|  | Cases (n=409) |  | Sensitivity (%) | Specificity (%) | Youden’s J | AUC (95% CI) | prAUC (95% CI) |
| Base | 37 |  | 95 | 83 | 0.78 | 0.93 (0.90 to 0.97) | 0.50 (0.35 to 0.64) |
| 2 | 5 |  | 80 | 82 | 0.62 | 0.77 (0.52 to 1.02) | 0.04 (0.01 to 0.09) |
| 2A | 3 |  | 67 | 92 | 0.59 | 0.70 (0.26 to 1.14) | 0.02 (0.00 to 0.07) |
| 2B | 2 |  | 100 | 83 | 0.83 | 0.87 (0.78 to 0.95) | 0.02 (0.01 to 0.05) |
| 3 | 32 |  | 97 | 83 | 0.80 | 0.94 (0.91 to 0.97) | 0.47 (0.31 to 0.65) |
| 3A | 16 |  | 88 | 88 | 0.76 | 0.92 (0.86 to 0.97) | 0.24 (0.11 to 0.40) |
| 3B | 16 |  | 94 | 84 | 0.78 | 0.93 (0.87 to 0.98) | 0.32 (0.12 to 0.49) |

|  |  |
| --- | --- |
| Mean weighted summary statistics where each column is multiplied by the number of cases and then divided by the total. This is applied to for cases matching ‘^(ao\_fibula\_|general\_Fibula)’, total of 7 measurements. | |
| Statisic | Mean |
| Sensitivity | 0.93 |
| Specificity | 0.84 |
| YoudenJ | 0.77 |
| AUC | 0.92 |
| prAUC | 0.39 |

## Tibia distal

|  |  |  |  |  |  |  |  |
| --- | --- | --- | --- | --- | --- | --- | --- |
| The outcome measures for the most important groups. Criterion based on Youden’s Index (Youden 1950; Aoki et al. 1997; Shapiro 1999; Greiner et al. 2000) defined as YI(c)=max\_{c}(Se(c)+Sp(c)-1). This is identical (from an optimization point of view) to the method that maximizes the sum of Sensitivity and Specificity (Albert and Harris 1987; Zweig and Campbell 1993) and to the criterion that maximizes concordance, wich is a monotone function of the AUC | | | | | | | |
|  |  |  | Measurements | | | | |
|  | Cases (n=409) |  | Sensitivity (%) | Specificity (%) | Youden’s J | AUC (95% CI) | prAUC (95% CI) |
| A | | | | | | | |
| Base | 14 |  | 100 | 89 | 0.89 | 0.93 (0.90 to 0.96) | 0.18 (0.09 to 0.29) |
| 1 | 8 |  | 100 | 88 | 0.88 | 0.92 (0.89 to 0.96) | 0.11 (0.04 to 0.20) |
| 11 | 8 |  | 100 | 89 | 0.89 | 0.92 (0.89 to 0.95) | 0.10 (0.03 to 0.18) |
| 2 | 4 |  | 100 | 87 | 0.87 | 0.93 (0.88 to 0.99) | 0.05 (0.00 to 0.12) |
| B | | | | | | | |
| Base | 8 |  | 67 | 84 | 0.51 | 0.76 (0.51 to 1.01) | 0.11 (0.01 to 0.34) |
| 1 | 8 |  | 50 | 98 | 0.48 | 0.77 (0.54 to 1.00) | 0.12 (0.01 to 0.37) |
| 11 | 6 |  | 75 | 75 | 0.50 | 0.78 (0.52 to 1.04) | 0.06 (0.00 to 0.22) |
| 12 | 2 |  | 100 | 79 | 0.79 | 0.87 (0.70 to 1.04) | 0.02 (0.01 to 0.08) |
| C | | | | | | | |
| Base | 16 |  | 94 | 90 | 0.84 | 0.95 (0.92 to 0.98) | 0.33 (0.17 to 0.48) |

|  |  |
| --- | --- |
| Mean weighted summary statistics where each column is multiplied by the number of cases and then divided by the total. This is applied to for cases matching ‘^(ao\_tibia\_distal\_)’, total of 9 measurements. | |
| Statisic | Mean |
| Sensitivity | 0.88 |
| Specificity | 0.88 |
| YoudenJ | 0.76 |
| AUC | 0.88 |
| prAUC | 0.16 |

## Tibia diaphysis

|  |  |  |  |  |  |  |  |
| --- | --- | --- | --- | --- | --- | --- | --- |
| The outcome measures for the most important groups. Criterion based on Youden’s Index (Youden 1950; Aoki et al. 1997; Shapiro 1999; Greiner et al. 2000) defined as YI(c)=max\_{c}(Se(c)+Sp(c)-1). This is identical (from an optimization point of view) to the method that maximizes the sum of Sensitivity and Specificity (Albert and Harris 1987; Zweig and Campbell 1993) and to the criterion that maximizes concordance, wich is a monotone function of the AUC | | | | | | | |
|  |  |  | Measurements | | | | |
|  | Cases (n=409) |  | Sensitivity (%) | Specificity (%) | Youden’s J | AUC (95% CI) | prAUC (95% CI) |
| Base | 27 |  | 100 | 89 | 0.89 | 0.97 (0.95 to 0.99) | 0.59 (0.39 to 0.74) |
| A | | | | | | | |
| Base | 16 |  | 100 | 81 | 0.81 | 0.94 (0.91 to 0.97) | 0.26 (0.12 to 0.41) |
| 1 | 16 |  | 100 | 85 | 0.85 | 0.94 (0.92 to 0.97) | 0.26 (0.13 to 0.42) |
| B | | | | | | | |
| Base | 7 |  | 100 | 84 | 0.84 | 0.95 (0.90 to 0.99) | 0.16 (0.05 to 0.32) |
| 2 | 7 |  | 100 | 85 | 0.85 | 0.94 (0.90 to 0.99) | 0.15 (0.04 to 0.33) |

|  |  |
| --- | --- |
| Mean weighted summary statistics where each column is multiplied by the number of cases and then divided by the total. This is applied to for cases matching ‘^(ao\_tibia\_diaphysis\_|general\_Tibia\_diaphyseal)’, total of 5 measurements. | |
| Statisic | Mean |
| Sensitivity | 1.00 |
| Specificity | 0.86 |
| YoudenJ | 0.86 |
| AUC | 0.95 |
| prAUC | 0.36 |

## Foot fractures

|  |  |  |  |  |  |  |  |
| --- | --- | --- | --- | --- | --- | --- | --- |
| The outcome measures for the most important groups. Criterion based on Youden’s Index (Youden 1950; Aoki et al. 1997; Shapiro 1999; Greiner et al. 2000) defined as YI(c)=max\_{c}(Se(c)+Sp(c)-1). This is identical (from an optimization point of view) to the method that maximizes the sum of Sensitivity and Specificity (Albert and Harris 1987; Zweig and Campbell 1993) and to the criterion that maximizes concordance, wich is a monotone function of the AUC | | | | | | | |
|  |  |  | Measurements | | | | |
|  | Cases (n=409) |  | Sensitivity (%) | Specificity (%) | Youden’s J | AUC (95% CI) | prAUC (95% CI) |
| Base | 40 |  | 94 | 76 | 0.71 | 0.92 (0.88 to 0.95) | 0.55 (0.34 to 0.70) |
| Calcaneus | 17 |  | 94 | 81 | 0.75 | 0.94 (0.89 to 0.98) | 0.49 (0.17 to 0.66) |
| Os metatarsale | 17 |  | 100 | 73 | 0.73 | 0.88 (0.83 to 0.93) | 0.13 (0.06 to 0.22) |
| Os metatarsale II | 2 |  | 100 | 88 | 0.88 | 0.90 (0.85 to 0.95) | 0.02 (0.01 to 0.07) |
| Os metatarsale V | 14 |  | 100 | 73 | 0.73 | 0.88 (0.82 to 0.94) | 0.10 (0.05 to 0.19) |
| Os naviculare | 3 |  | 100 | 85 | 0.85 | 0.92 (0.78 to 1.07) | 0.10 (0.01 to 0.38) |
| Talus | 4 |  | 67 | 95 | 0.61 | 0.81 (0.54 to 1.09) | 0.04 (0.00 to 0.13) |

|  |  |
| --- | --- |
| Mean weighted summary statistics where each column is multiplied by the number of cases and then divided by the total. This is applied to for cases matching ‘^(foot\_bones\_|general\_Foot)’, total of 7 measurements. | |
| Statisic | Mean |
| Sensitivity | 0.95 |
| Specificity | 0.77 |
| YoudenJ | 0.73 |
| AUC | 0.90 |
| prAUC | 0.36 |

# Inter-rater results
