# Supplementary material for: External validation of an artificial intelligence multi-label deep learning model capable of ankle fracture classification
Source: BMC Musculoskelet Disord. 2024 Oct 4;25:788. doi: 10.1186/s12891-024-07884-2 (PMC11451058; doi:10.1186/s12891-024-07884-2)
Supplement: Supplementary file 3 — Supplementary Material 3. [file 12891_2024_7884_MOESM3_ESM.html]

 

 

 

 
 
 


 Eternal ankle fracture analysis 

 
 
 
 
 
 
 
 
 
 
 

 

 
 


 


 

 

 


 

 


 


 


 Eternal ankle fracture analysis 

 

 
 
  Results 
 
  Per worker
outcome  
  Consensus
outcomes 
 
  Malleolar fractures  
  Fibula
fractures  
  
  Max
Gordon’s analysis 
 
  Malleolar fractures  
  Fibula
fractures  
  
  Swedish test set outcomes 
 
  Malleolar fractures  
  Fibula
fractures  
  
  Inter-rater agreements 
 
  ICC  
  Kappa values for Frank &
Max  
  
  
  Image analysis 
 
  Malloelar 
 
  A-class  
  B-class  
  C-class  
  
  
 
 

 
 Results 
 
 
 
 
 
 
Yes
 
 
No
 
 
 
 
 
 
General
 
 
 
 
Fracture
 
 
 
 
  base
 
 
277 (69.4%)
 
 
122 (30.6%)
 
 
 
 
  Malleolar
 
 
274 (68.7%)
 
 
125 (31.3%)
 
 
 
 
  Fibula
 
 
3 (0.8%)
 
 
396 (99.2%)
 
 
 
 
  Foot
 
 
2 (0.5%)
 
 
397 (99.5%)
 
 
 
 
Previous
 
 
15 (3.8%)
 
 
384 (96.2%)
 
 
 
 
Malleolar
 
 
 
 
A
 
 
 
 
  base
 
 
94 (23.6%)
 
 
305 (76.4%)
 
 
 
 
  1
 
 
93 (23.3%)
 
 
306 (76.7%)
 
 
 
 
  …1
 
 
5 (1.3%)
 
 
394 (98.7%)
 
 
 
 
  …2
 
 
28 (7.0%)
 
 
371 (93.0%)
 
 
 
 
  …3
 
 
60 (15.0%)
 
 
339 (85.0%)
 
 
 
 
  2
 
 
1 (0.3%)
 
 
398 (99.7%)
 
 
 
 
  …2
 
 
1 (0.3%)
 
 
398 (99.7%)
 
 
 
 
B
 
 
 
 
  base
 
 
142 (35.6%)
 
 
257 (64.4%)
 
 
 
 
  1
 
 
116 (29.1%)
 
 
283 (70.9%)
 
 
 
 
  …1
 
 
87 (21.8%)
 
 
312 (78.2%)
 
 
 
 
  …2
 
 
27 (6.8%)
 
 
372 (93.2%)
 
 
 
 
  …3
 
 
2 (0.5%)
 
 
397 (99.5%)
 
 
 
 
  2
 
 
21 (5.3%)
 
 
378 (94.7%)
 
 
 
 
  …1
 
 
18 (4.5%)
 
 
381 (95.5%)
 
 
 
 
  …2
 
 
3 (0.8%)
 
 
396 (99.2%)
 
 
 
 
  3
 
 
5 (1.3%)
 
 
394 (98.7%)
 
 
 
 
  …1
 
 
5 (1.3%)
 
 
394 (98.7%)
 
 
 
 
C
 
 
 
 
  base
 
 
38 (9.5%)
 
 
361 (90.5%)
 
 
 
 
  1
 
 
28 (7.0%)
 
 
371 (93.0%)
 
 
 
 
  …1
 
 
27 (6.8%)
 
 
372 (93.2%)
 
 
 
 
  …2
 
 
1 (0.3%)
 
 
398 (99.7%)
 
 
 
 
  2
 
 
9 (2.3%)
 
 
390 (97.7%)
 
 
 
 
  …1
 
 
9 (2.3%)
 
 
390 (97.7%)
 
 
 
 
  3
 
 
1 (0.3%)
 
 
398 (99.7%)
 
 
 
 
  …1
 
 
1 (0.3%)
 
 
398 (99.7%)
 
 
 
 
Fibula
 
 
 
 
2
 
 
 
 
  base
 
 
0 (0.0%)
 
 
399 (100.0%)
 
 
 
 
  A
 
 
0 (0.0%)
 
 
399 (100.0%)
 
 
 
 
  …→c
 
 
0 (0.0%)
 
 
399 (100.0%)
 
 
 
 
3
 
 
 
 
  base
 
 
3 (0.8%)
 
 
396 (99.2%)
 
 
 
 
  A
 
 
2 (0.5%)
 
 
397 (99.5%)
 
 
 
 
  B
 
 
1 (0.3%)
 
 
398 (99.7%)
 
 
 
 
 
Table 1
 
 
 
 
 Per worker outcome 
 This is mostly for understanding the biases of each classifier and
not really for reporting. 
 
 
 
 
 
 
Frank
 
 
 
 
 
 
Job
 
 
 
 
 
 
Ehsan
 
 
 
 
 
 
Max
 
 
 
 
 
 
Yes
 
 
No
 
 
 
 
 
 
Yes
 
 
No
 
 
 
 
 
 
Yes
 
 
No
 
 
 
 
 
 
Yes
 
 
No
 
 
 
 
 
 
General
 
 
 
 
Fracture
 
 
 
 
  base
 
 
278 (69.7%)
 
 
121 (30.3%)
 
 
 
 
 
 
83 (83.0%)
 
 
17 (17.0%)
 
 
 
 
 
 
202 (67.6%)
 
 
97 (32.4%)
 
 
 
 
 
 
276 (69.2%)
 
 
123 (30.8%)
 
 
 
 
  Malleolar
 
 
278 (69.7%)
 
 
121 (30.3%)
 
 
 
 
 
 
83 (83.0%)
 
 
17 (17.0%)
 
 
 
 
 
 
194 (64.9%)
 
 
105 (35.1%)
 
 
 
 
 
 
271 (67.9%)
 
 
128 (32.1%)
 
 
 
 
  Fibula
 
 
0 (0.0%)
 
 
399 (100.0%)
 
 
 
 
 
 
1 (1.0%)
 
 
99 (99.0%)
 
 
 
 
 
 
108 (36.1%)
 
 
191 (63.9%)
 
 
 
 
 
 
5 (1.3%)
 
 
394 (98.7%)
 
 
 
 
  Foot
 
 
0 (0.0%)
 
 
399 (100.0%)
 
 
 
 
 
 
0 (0.0%)
 
 
100 (100.0%)
 
 
 
 
 
 
3 (1.0%)
 
 
296 (99.0%)
 
 
 
 
 
 
2 (0.5%)
 
 
397 (99.5%)
 
 
 
 
Previous
 
 
13 (3.3%)
 
 
386 (96.7%)
 
 
 
 
 
 
0 (0.0%)
 
 
100 (100.0%)
 
 
 
 
 
 
15 (5.0%)
 
 
284 (95.0%)
 
 
 
 
 
 
8 (2.0%)
 
 
391 (98.0%)
 
 
 
 
Malleolar
 
 
 
 
A
 
 
 
 
  base
 
 
101 (25.3%)
 
 
298 (74.7%)
 
 
 
 
 
 
28 (28.0%)
 
 
72 (72.0%)
 
 
 
 
 
 
64 (21.4%)
 
 
235 (78.6%)
 
 
 
 
 
 
95 (23.8%)
 
 
304 (76.2%)
 
 
 
 
  1
 
 
100 (25.1%)
 
 
299 (74.9%)
 
 
 
 
 
 
27 (27.0%)
 
 
73 (73.0%)
 
 
 
 
 
 
64 (21.4%)
 
 
235 (78.6%)
 
 
 
 
 
 
92 (23.1%)
 
 
307 (76.9%)
 
 
 
 
  …1
 
 
6 (1.5%)
 
 
393 (98.5%)
 
 
 
 
 
 
0 (0.0%)
 
 
100 (100.0%)
 
 
 
 
 
 
10 (3.3%)
 
 
289 (96.7%)
 
 
 
 
 
 
9 (2.3%)
 
 
390 (97.7%)
 
 
 
 
  …2
 
 
29 (7.3%)
 
 
370 (92.7%)
 
 
 
 
 
 
7 (7.0%)
 
 
93 (93.0%)
 
 
 
 
 
 
31 (10.4%)
 
 
268 (89.6%)
 
 
 
 
 
 
24 (6.0%)
 
 
375 (94.0%)
 
 
 
 
  …3
 
 
65 (16.3%)
 
 
334 (83.7%)
 
 
 
 
 
 
19 (19.0%)
 
 
81 (81.0%)
 
 
 
 
 
 
23 (7.7%)
 
 
276 (92.3%)
 
 
 
 
 
 
59 (14.8%)
 
 
340 (85.2%)
 
 
 
 
  2
 
 
1 (0.3%)
 
 
398 (99.7%)
 
 
 
 
 
 
0 (0.0%)
 
 
100 (100.0%)
 
 
 
 
 
 
0 (0.0%)
 
 
299 (100.0%)
 
 
 
 
 
 
3 (0.8%)
 
 
396 (99.2%)
 
 
 
 
  …2
 
 
1 (0.3%)
 
 
398 (99.7%)
 
 
 
 
 
 
0 (0.0%)
 
 
100 (100.0%)
 
 
 
 
 
 
0 (0.0%)
 
 
299 (100.0%)
 
 
 
 
 
 
2 (0.5%)
 
 
397 (99.5%)
 
 
 
 
  …3
 
 
0 (0.0%)
 
 
399 (100.0%)
 
 
 
 
 
 
0 (0.0%)
 
 
100 (100.0%)
 
 
 
 
 
 
0 (0.0%)
 
 
299 (100.0%)
 
 
 
 
 
 
1 (0.3%)
 
 
398 (99.7%)
 
 
 
 
  3
 
 
0 (0.0%)
 
 
399 (100.0%)
 
 
 
 
 
 
1 (1.0%)
 
 
99 (99.0%)
 
 
 
 
 
 
0 (0.0%)
 
 
299 (100.0%)
 
 
 
 
 
 
0 (0.0%)
 
 
399 (100.0%)
 
 
 
 
  …2
 
 
0 (0.0%)
 
 
399 (100.0%)
 
 
 
 
 
 
1 (1.0%)
 
 
99 (99.0%)
 
 
 
 
 
 
0 (0.0%)
 
 
299 (100.0%)
 
 
 
 
 
 
0 (0.0%)
 
 
399 (100.0%)
 
 
 
 
B
 
 
 
 
  base
 
 
142 (35.6%)
 
 
257 (64.4%)
 
 
 
 
 
 
41 (41.0%)
 
 
59 (59.0%)
 
 
 
 
 
 
111 (37.1%)
 
 
188 (62.9%)
 
 
 
 
 
 
146 (36.6%)
 
 
253 (63.4%)
 
 
 
 
  1
 
 
129 (32.3%)
 
 
270 (67.7%)
 
 
 
 
 
 
34 (34.0%)
 
 
66 (66.0%)
 
 
 
 
 
 
79 (26.4%)
 
 
220 (73.6%)
 
 
 
 
 
 
123 (30.8%)
 
 
276 (69.2%)
 
 
 
 
  …1
 
 
73 (18.3%)
 
 
326 (81.7%)
 
 
 
 
 
 
28 (28.0%)
 
 
72 (72.0%)
 
 
 
 
 
 
51 (17.1%)
 
 
248 (82.9%)
 
 
 
 
 
 
89 (22.3%)
 
 
310 (77.7%)
 
 
 
 
  …2
 
 
53 (13.3%)
 
 
346 (86.7%)
 
 
 
 
 
 
1 (1.0%)
 
 
99 (99.0%)
 
 
 
 
 
 
17 (5.7%)
 
 
282 (94.3%)
 
 
 
 
 
 
32 (8.0%)
 
 
367 (92.0%)
 
 
 
 
  …3
 
 
3 (0.8%)
 
 
396 (99.2%)
 
 
 
 
 
 
2 (2.0%)
 
 
98 (98.0%)
 
 
 
 
 
 
11 (3.7%)
 
 
288 (96.3%)
 
 
 
 
 
 
2 (0.5%)
 
 
397 (99.5%)
 
 
 
 
  2
 
 
13 (3.3%)
 
 
386 (96.7%)
 
 
 
 
 
 
7 (7.0%)
 
 
93 (93.0%)
 
 
 
 
 
 
27 (9.0%)
 
 
272 (91.0%)
 
 
 
 
 
 
20 (5.0%)
 
 
379 (95.0%)
 
 
 
 
  …1
 
 
13 (3.3%)
 
 
386 (96.7%)
 
 
 
 
 
 
6 (6.0%)
 
 
94 (94.0%)
 
 
 
 
 
 
23 (7.7%)
 
 
276 (92.3%)
 
 
 
 
 
 
16 (4.0%)
 
 
383 (96.0%)
 
 
 
 
  …2
 
 
0 (0.0%)
 
 
399 (100.0%)
 
 
 
 
 
 
0 (0.0%)
 
 
100 (100.0%)
 
 
 
 
 
 
2 (0.7%)
 
 
297 (99.3%)
 
 
 
 
 
 
3 (0.8%)
 
 
396 (99.2%)
 
 
 
 
  …3
 
 
0 (0.0%)
 
 
399 (100.0%)
 
 
 
 
 
 
1 (1.0%)
 
 
99 (99.0%)
 
 
 
 
 
 
2 (0.7%)
 
 
297 (99.3%)
 
 
 
 
 
 
1 (0.3%)
 
 
398 (99.7%)
 
 
 
 
  3
 
 
0 (0.0%)
 
 
399 (100.0%)
 
 
 
 
 
 
0 (0.0%)
 
 
100 (100.0%)
 
 
 
 
 
 
5 (1.7%)
 
 
294 (98.3%)
 
 
 
 
 
 
3 (0.8%)
 
 
396 (99.2%)
 
 
 
 
  …1
 
 
0 (0.0%)
 
 
399 (100.0%)
 
 
 
 
 
 
0 (0.0%)
 
 
100 (100.0%)
 
 
 
 
 
 
4 (1.3%)
 
 
295 (98.7%)
 
 
 
 
 
 
3 (0.8%)
 
 
396 (99.2%)
 
 
 
 
  …3
 
 
0 (0.0%)
 
 
399 (100.0%)
 
 
 
 
 
 
0 (0.0%)
 
 
100 (100.0%)
 
 
 
 
 
 
1 (0.3%)
 
 
298 (99.7%)
 
 
 
 
 
 
0 (0.0%)
 
 
399 (100.0%)
 
 
 
 
C
 
 
 
 
  base
 
 
35 (8.8%)
 
 
364 (91.2%)
 
 
 
 
 
 
14 (14.0%)
 
 
86 (86.0%)
 
 
 
 
 
 
19 (6.4%)
 
 
280 (93.6%)
 
 
 
 
 
 
30 (7.5%)
 
 
369 (92.5%)
 
 
 
 
  1
 
 
30 (7.5%)
 
 
369 (92.5%)
 
 
 
 
 
 
9 (9.0%)
 
 
91 (91.0%)
 
 
 
 
 
 
8 (2.7%)
 
 
291 (97.3%)
 
 
 
 
 
 
22 (5.5%)
 
 
377 (94.5%)
 
 
 
 
  …1
 
 
29 (7.3%)
 
 
370 (92.7%)
 
 
 
 
 
 
1 (1.0%)
 
 
99 (99.0%)
 
 
 
 
 
 
8 (2.7%)
 
 
291 (97.3%)
 
 
 
 
 
 
22 (5.5%)
 
 
377 (94.5%)
 
 
 
 
  …2
 
 
1 (0.3%)
 
 
398 (99.7%)
 
 
 
 
 
 
1 (1.0%)
 
 
99 (99.0%)
 
 
 
 
 
 
0 (0.0%)
 
 
299 (100.0%)
 
 
 
 
 
 
0 (0.0%)
 
 
399 (100.0%)
 
 
 
 
  2
 
 
4 (1.0%)
 
 
395 (99.0%)
 
 
 
 
 
 
5 (5.0%)
 
 
95 (95.0%)
 
 
 
 
 
 
10 (3.3%)
 
 
289 (96.7%)
 
 
 
 
 
 
8 (2.0%)
 
 
391 (98.0%)
 
 
 
 
  …1
 
 
4 (1.0%)
 
 
395 (99.0%)
 
 
 
 
 
 
1 (1.0%)
 
 
99 (99.0%)
 
 
 
 
 
 
7 (2.3%)
 
 
292 (97.7%)
 
 
 
 
 
 
8 (2.0%)
 
 
391 (98.0%)
 
 
 
 
  3
 
 
1 (0.3%)
 
 
398 (99.7%)
 
 
 
 
 
 
0 (0.0%)
 
 
100 (100.0%)
 
 
 
 
 
 
1 (0.3%)
 
 
298 (99.7%)
 
 
 
 
 
 
0 (0.0%)
 
 
399 (100.0%)
 
 
 
 
  …1
 
 
1 (0.3%)
 
 
398 (99.7%)
 
 
 
 
 
 
0 (0.0%)
 
 
100 (100.0%)
 
 
 
 
 
 
0 (0.0%)
 
 
299 (100.0%)
 
 
 
 
 
 
0 (0.0%)
 
 
399 (100.0%)
 
 
 
 
  …2
 
 
0 (0.0%)
 
 
399 (100.0%)
 
 
 
 
 
 
0 (0.0%)
 
 
100 (100.0%)
 
 
 
 
 
 
1 (0.3%)
 
 
298 (99.7%)
 
 
 
 
 
 
0 (0.0%)
 
 
399 (100.0%)
 
 
 
 
Fibula
 
 
 
 
2
 
 
 
 
  base
 
 
0 (0.0%)
 
 
399 (100.0%)
 
 
 
 
 
 
0 (0.0%)
 
 
100 (100.0%)
 
 
 
 
 
 
11 (3.7%)
 
 
288 (96.3%)
 
 
 
 
 
 
1 (0.3%)
 
 
398 (99.7%)
 
 
 
 
  A
 
 
0 (0.0%)
 
 
399 (100.0%)
 
 
 
 
 
 
0 (0.0%)
 
 
100 (100.0%)
 
 
 
 
 
 
5 (1.7%)
 
 
294 (98.3%)
 
 
 
 
 
 
1 (0.3%)
 
 
398 (99.7%)
 
 
 
 
  B
 
 
0 (0.0%)
 
 
399 (100.0%)
 
 
 
 
 
 
0 (0.0%)
 
 
100 (100.0%)
 
 
 
 
 
 
6 (2.0%)
 
 
293 (98.0%)
 
 
 
 
 
 
0 (0.0%)
 
 
399 (100.0%)
 
 
 
 
  …→b
 
 
0 (0.0%)
 
 
399 (100.0%)
 
 
 
 
 
 
0 (0.0%)
 
 
100 (100.0%)
 
 
 
 
 
 
3 (1.0%)
 
 
296 (99.0%)
 
 
 
 
 
 
0 (0.0%)
 
 
399 (100.0%)
 
 
 
 
  …→c
 
 
0 (0.0%)
 
 
399 (100.0%)
 
 
 
 
 
 
0 (0.0%)
 
 
100 (100.0%)
 
 
 
 
 
 
8 (2.7%)
 
 
291 (97.3%)
 
 
 
 
 
 
1 (0.3%)
 
 
398 (99.7%)
 
 
 
 
3
 
 
 
 
  base
 
 
0 (0.0%)
 
 
399 (100.0%)
 
 
 
 
 
 
1 (1.0%)
 
 
99 (99.0%)
 
 
 
 
 
 
97 (32.4%)
 
 
202 (67.6%)
 
 
 
 
 
 
4 (1.0%)
 
 
395 (99.0%)
 
 
 
 
  A
 
 
0 (0.0%)
 
 
399 (100.0%)
 
 
 
 
 
 
0 (0.0%)
 
 
100 (100.0%)
 
 
 
 
 
 
85 (28.4%)
 
 
214 (71.6%)
 
 
 
 
 
 
2 (0.5%)
 
 
397 (99.5%)
 
 
 
 
  B
 
 
0 (0.0%)
 
 
399 (100.0%)
 
 
 
 
 
 
0 (0.0%)
 
 
100 (100.0%)
 
 
 
 
 
 
12 (4.0%)
 
 
287 (96.0%)
 
 
 
 
 
 
2 (0.5%)
 
 
397 (99.5%)
 
 
 
 
 
 
 Consensus outcomes 
 
 Malleolar fractures 
 
 
 
 
 
 
Cases (n=399)
 
 
Sensitivity (%)
 
 
Specificity (%)
 
 
Youden’s J
 
 
AUC (95% CI)
 
 
PR-AUC (95% CI)
 
 
 
 
 
 
General
 
 
274
 
 
74
 
 
88
 
 
0.62
 
 
0.86 (0.82 to 0.89)
 
 
0.93 (0.91 to 0.96)
 
 
 
 
A
 
 
 
 
  Base
 
 
94
 
 
66
 
 
69
 
 
0.35
 
 
0.74 (0.68 to 0.80)
 
 
0.52 (0.43 to 0.61)
 
 
 
 
  1
 
 
93
 
 
55
 
 
82
 
 
0.37
 
 
0.75 (0.69 to 0.81)
 
 
0.57 (0.46 to 0.64)
 
 
 
 
  …1
 
 
5
 
 
40
 
 
96
 
 
0.36
 
 
0.63 (0.33 to 0.94)
 
 
0.04 (0.00 to 0.16)
 
 
 
 
  …2
 
 
28
 
 
86
 
 
60
 
 
0.46
 
 
0.78 (0.69 to 0.87)
 
 
0.26 (0.11 to 0.39)
 
 
 
 
  …3
 
 
60
 
 
52
 
 
78
 
 
0.30
 
 
0.68 (0.61 to 0.76)
 
 
0.30 (0.21 to 0.42)
 
 
 
 
B
 
 
 
 
  Base
 
 
142
 
 
83
 
 
81
 
 
0.64
 
 
0.90 (0.87 to 0.93)
 
 
0.84 (0.78 to 0.88)
 
 
 
 
  1
 
 
116
 
 
80
 
 
74
 
 
0.54
 
 
0.84 (0.80 to 0.88)
 
 
0.68 (0.60 to 0.76)
 
 
 
 
  …1
 
 
87
 
 
86
 
 
66
 
 
0.52
 
 
0.80 (0.75 to 0.85)
 
 
0.47 (0.37 to 0.57)
 
 
 
 
  …2
 
 
27
 
 
89
 
 
62
 
 
0.51
 
 
0.80 (0.72 to 0.88)
 
 
0.19 (0.11 to 0.31)
 
 
 
 
  …3
 
 
2
 
 
100
 
 
38
 
 
0.38
 
 
0.60 (0.17 to 1.02)
 
 
0.01 (0.00 to 0.02)
 
 
 
 
  2
 
 
21
 
 
71
 
 
88
 
 
0.59
 
 
0.85 (0.75 to 0.94)
 
 
0.32 (0.18 to 0.49)
 
 
 
 
  …1
 
 
18
 
 
72
 
 
87
 
 
0.60
 
 
0.85 (0.75 to 0.95)
 
 
0.33 (0.16 to 0.56)
 
 
 
 
  …2
 
 
3
 
 
100
 
 
89
 
 
0.89
 
 
0.93 (0.88 to 0.99)
 
 
0.05 (0.01 to 0.16)
 
 
 
 
  3
 
 
5
 
 
60
 
 
99
 
 
0.59
 
 
0.82 (0.61 to 1.04)
 
 
0.19 (0.01 to 0.47)
 
 
 
 
  …1
 
 
5
 
 
60
 
 
96
 
 
0.56
 
 
0.82 (0.63 to 1.02)
 
 
0.12 (0.01 to 0.30)
 
 
 
 
C
 
 
 
 
  Base
 
 
38
 
 
82
 
 
90
 
 
0.72
 
 
0.89 (0.82 to 0.96)
 
 
0.63 (0.45 to 0.77)
 
 
 
 
  1
 
 
28
 
 
89
 
 
85
 
 
0.74
 
 
0.90 (0.84 to 0.96)
 
 
0.42 (0.26 to 0.62)
 
 
 
 
  …1
 
 
27
 
 
85
 
 
87
 
 
0.73
 
 
0.90 (0.84 to 0.97)
 
 
0.44 (0.22 to 0.64)
 
 
 
 
  2
 
 
9
 
 
89
 
 
89
 
 
0.78
 
 
0.92 (0.82 to 1.01)
 
 
0.19 (0.04 to 0.37)
 
 
 
 
  …1
 
 
9
 
 
89
 
 
91
 
 
0.80
 
 
0.90 (0.79 to 1.02)
 
 
0.16 (0.05 to 0.31)
 
 
 
 
A11 or A12
 
 
33
 
 
76
 
 
68
 
 
0.44
 
 
0.77 (0.69 to 0.85)
 
 
0.29 (0.16 to 0.40)
 
 
 
 
A12 or A13
 
 
88
 
 
69
 
 
70
 
 
0.39
 
 
0.74 (0.68 to 0.80)
 
 
0.54 (0.44 to 0.62)
 
 
 
 
B11 or B12
 
 
29
 
 
86
 
 
65
 
 
0.51
 
 
0.79 (0.71 to 0.86)
 
 
0.19 (0.13 to 0.30)
 
 
 
 
B11 or B12 or B21
 
 
47
 
 
85
 
 
68
 
 
0.53
 
 
0.82 (0.76 to 0.88)
 
 
0.32 (0.22 to 0.43)
 
 
 
 
medial malleolus
 
 
4
 
 
100
 
 
39
 
 
0.39
 
 
0.64 (0.38 to 0.89)
 
 
0.01 (0.00 to 0.04)
 
 
 
 
 
The outcome measures for the most important groups. Criterion based on
Youden’s Index (Youden 1950; Aoki et al. 1997; Shapiro 1999; Greiner et
al. 2000) defined as YI(c)=max_{c}(Se(c)+Sp(c)-1). This is identical
(from an optimization point of view) to the method that maximizes the
sum of Sensitivity and Specificity (Albert and Harris 1987; Zweig and
Campbell 1993) and to the criterion that maximizes concordance, wich is
a monotone function of the AUC
 
 
 
 
 
 
 
Statisic
 
 
Mean
 
 
 
 
 
 
Sensitivity
 
 
0.75
 
 
 
 
Specificity
 
 
0.78
 
 
 
 
YoudenJ
 
 
0.53
 
 
 
 
AUC
 
 
0.82
 
 
 
 
prAUC
 
 
0.59
 
 
 
 
 
Mean weighted summary statistics where each column is multiplied by the
number of cases and then divided by the total. This is applied to for
cases matching
‘^(ao|alt_ao) malleolar|^general (Malleolar|Fracture)$’, total of
30 measurements.
 
 
 
 
 
 Fibula fractures 
 
 
 
 
 
 
Cases (n=399)
 
 
Sensitivity (%)
 
 
Specificity (%)
 
 
Youden’s J
 
 
AUC (95% CI)
 
 
PR-AUC (95% CI)
 
 
 
 
 
 
General
 
 
3
 
 
100
 
 
63
 
 
0.63
 
 
0.86 (0.63 to 1.09)
 
 
0.06 (0.00 to 0.21)
 
 
 
 
3
 
 
3
 
 
100
 
 
61
 
 
0.61
 
 
0.79 (0.60 to 0.97)
 
 
0.02 (0.00 to 0.05)
 
 
 
 
3A
 
 
2
 
 
100
 
 
55
 
 
0.55
 
 
0.71 (0.39 to 1.02)
 
 
0.01 (0.00 to 0.03)
 
 
 
 
 
The outcome measures for the most important groups. Criterion based on
Youden’s Index (Youden 1950; Aoki et al. 1997; Shapiro 1999; Greiner et
al. 2000) defined as YI(c)=max_{c}(Se(c)+Sp(c)-1). This is identical
(from an optimization point of view) to the method that maximizes the
sum of Sensitivity and Specificity (Albert and Harris 1987; Zweig and
Campbell 1993) and to the criterion that maximizes concordance, wich is
a monotone function of the AUC
 
 
 
 
 
 
 Max Gordon’s analysis 
 
 Malleolar fractures 
 
 
 
 
 
 
Cases (n=399)
 
 
Sensitivity (%)
 
 
Specificity (%)
 
 
Youden’s J
 
 
AUC (95% CI)
 
 
PR-AUC (95% CI)
 
 
 
 
 
 
General
 
 
271
 
 
78
 
 
83
 
 
0.61
 
 
0.85 (0.81 to 0.89)
 
 
0.92 (0.89 to 0.94)
 
 
 
 
A
 
 
 
 
  Base
 
 
95
 
 
61
 
 
76
 
 
0.37
 
 
0.74 (0.69 to 0.80)
 
 
0.52 (0.41 to 0.61)
 
 
 
 
  1
 
 
92
 
 
72
 
 
67
 
 
0.39
 
 
0.75 (0.70 to 0.81)
 
 
0.55 (0.43 to 0.66)
 
 
 
 
  …1
 
 
9
 
 
56
 
 
90
 
 
0.46
 
 
0.75 (0.57 to 0.93)
 
 
0.08 (0.01 to 0.19)
 
 
 
 
  …2
 
 
24
 
 
88
 
 
60
 
 
0.48
 
 
0.77 (0.68 to 0.87)
 
 
0.19 (0.08 to 0.31)
 
 
 
 
  …3
 
 
59
 
 
53
 
 
78
 
 
0.30
 
 
0.68 (0.61 to 0.76)
 
 
0.30 (0.20 to 0.43)
 
 
 
 
  2
 
 
3
 
 
100
 
 
50
 
 
0.50
 
 
0.74 (0.47 to 1.00)
 
 
0.02 (0.00 to 0.10)
 
 
 
 
B
 
 
 
 
  Base
 
 
146
 
 
89
 
 
76
 
 
0.65
 
 
0.90 (0.87 to 0.93)
 
 
0.84 (0.78 to 0.89)
 
 
 
 
  1
 
 
123
 
 
82
 
 
76
 
 
0.59
 
 
0.85 (0.81 to 0.89)
 
 
0.71 (0.62 to 0.78)
 
 
 
 
  …1
 
 
89
 
 
88
 
 
67
 
 
0.54
 
 
0.81 (0.76 to 0.85)
 
 
0.46 (0.35 to 0.56)
 
 
 
 
  …2
 
 
32
 
 
84
 
 
65
 
 
0.49
 
 
0.79 (0.72 to 0.87)
 
 
0.25 (0.12 to 0.36)
 
 
 
 
  …3
 
 
2
 
 
100
 
 
38
 
 
0.38
 
 
0.38 (0.34 to 0.43)
 
 
0.00 (0.00 to 0.01)
 
 
 
 
  2
 
 
20
 
 
75
 
 
90
 
 
0.65
 
 
0.88 (0.81 to 0.96)
 
 
0.43 (0.16 to 0.62)
 
 
 
 
  …1
 
 
16
 
 
69
 
 
95
 
 
0.64
 
 
0.88 (0.80 to 0.96)
 
 
0.29 (0.13 to 0.47)
 
 
 
 
  …2
 
 
3
 
 
100
 
 
89
 
 
0.89
 
 
0.96 (0.89 to 1.02)
 
 
0.11 (0.01 to 0.36)
 
 
 
 
  3
 
 
3
 
 
100
 
 
41
 
 
0.41
 
 
0.62 (0.26 to 0.98)
 
 
0.03 (0.00 to 0.19)
 
 
 
 
  …1
 
 
3
 
 
33
 
 
96
 
 
0.29
 
 
0.59 (0.19 to 0.98)
 
 
0.02 (0.00 to 0.08)
 
 
 
 
C
 
 
 
 
  Base
 
 
30
 
 
93
 
 
85
 
 
0.78
 
 
0.94 (0.88 to 0.99)
 
 
0.64 (0.46 to 0.79)
 
 
 
 
  1
 
 
22
 
 
91
 
 
84
 
 
0.74
 
 
0.92 (0.87 to 0.97)
 
 
0.39 (0.22 to 0.59)
 
 
 
 
  …1
 
 
22
 
 
95
 
 
78
 
 
0.73
 
 
0.92 (0.87 to 0.97)
 
 
0.43 (0.23 to 0.60)
 
 
 
 
  2
 
 
8
 
 
100
 
 
89
 
 
0.89
 
 
0.96 (0.94 to 0.99)
 
 
0.21 (0.06 to 0.39)
 
 
 
 
  …1
 
 
8
 
 
100
 
 
91
 
 
0.91
 
 
0.96 (0.94 to 0.98)
 
 
0.18 (0.06 to 0.35)
 
 
 
 
A11 or A12
 
 
33
 
 
79
 
 
68
 
 
0.47
 
 
0.77 (0.69 to 0.86)
 
 
0.27 (0.12 to 0.42)
 
 
 
 
A12 or A13
 
 
83
 
 
71
 
 
70
 
 
0.41
 
 
0.73 (0.67 to 0.80)
 
 
0.47 (0.34 to 0.57)
 
 
 
 
B11 or B12
 
 
34
 
 
82
 
 
66
 
 
0.48
 
 
0.78 (0.70 to 0.85)
 
 
0.24 (0.14 to 0.37)
 
 
 
 
B11 or B12 or B21
 
 
50
 
 
84
 
 
68
 
 
0.52
 
 
0.82 (0.76 to 0.87)
 
 
0.36 (0.24 to 0.51)
 
 
 
 
medial malleolus
 
 
7
 
 
57
 
 
89
 
 
0.46
 
 
0.75 (0.55 to 0.94)
 
 
0.07 (0.01 to 0.19)
 
 
 
 
 
The outcome measures for the most important groups. Criterion based on
Youden’s Index (Youden 1950; Aoki et al. 1997; Shapiro 1999; Greiner et
al. 2000) defined as YI(c)=max_{c}(Se(c)+Sp(c)-1). This is identical
(from an optimization point of view) to the method that maximizes the
sum of Sensitivity and Specificity (Albert and Harris 1987; Zweig and
Campbell 1993) and to the criterion that maximizes concordance, wich is
a monotone function of the AUC
 
 
 
 
 
 
 
Statisic
 
 
Mean
 
 
 
 
 
 
Sensitivity
 
 
0.79
 
 
 
 
Specificity
 
 
0.76
 
 
 
 
YoudenJ
 
 
0.54
 
 
 
 
AUC
 
 
0.82
 
 
 
 
prAUC
 
 
0.59
 
 
 
 
 
Mean weighted summary statistics where each column is multiplied by the
number of cases and then divided by the total. This is applied to for
cases matching
‘^(ao|alt_ao) malleolar|^general (Malleolar|Fracture)$’, total of
29 measurements.
 
 
 
 
 
 Fibula fractures 
 
 
 
 
 
 
Cases (n=399)
 
 
Sensitivity (%)
 
 
Specificity (%)
 
 
Youden’s J
 
 
AUC (95% CI)
 
 
PR-AUC (95% CI)
 
 
 
 
 
 
General
 
 
5
 
 
100
 
 
63
 
 
0.63
 
 
0.86 (0.74 to 0.99)
 
 
0.06 (0.01 to 0.18)
 
 
 
 
3
 
 
4
 
 
100
 
 
61
 
 
0.61
 
 
0.76 (0.61 to 0.90)
 
 
0.02 (0.00 to 0.05)
 
 
 
 
3A
 
 
2
 
 
100
 
 
55
 
 
0.55
 
 
0.71 (0.39 to 1.02)
 
 
0.01 (0.00 to 0.04)
 
 
 
 
3B
 
 
2
 
 
100
 
 
53
 
 
0.53
 
 
0.74 (0.32 to 1.15)
 
 
0.01 (0.00 to 0.08)
 
 
 
 
 
The outcome measures for the most important groups. Criterion based on
Youden’s Index (Youden 1950; Aoki et al. 1997; Shapiro 1999; Greiner et
al. 2000) defined as YI(c)=max_{c}(Se(c)+Sp(c)-1). This is identical
(from an optimization point of view) to the method that maximizes the
sum of Sensitivity and Specificity (Albert and Harris 1987; Zweig and
Campbell 1993) and to the criterion that maximizes concordance, wich is
a monotone function of the AUC
 
 
 
 
 
 
 Swedish test set outcomes 
 
 Malleolar fractures 
 
 
 
 
 
 
Cases (n=409)
 
 
Sensitivity (%)
 
 
Specificity (%)
 
 
Youden’s J
 
 
AUC (95% CI)
 
 
PR-AUC (95% CI)
 
 
 
 
 
 
General
 
 
216
 
 
86
 
 
93
 
 
0.79
 
 
0.95 (0.94 to 0.97)
 
 
0.96 (0.94 to 0.97)
 
 
 
 
A
 
 
 
 
  Base
 
 
32
 
 
78
 
 
76
 
 
0.54
 
 
0.84 (0.76 to 0.92)
 
 
0.46 (0.25 to 0.61)
 
 
 
 
  1
 
 
22
 
 
100
 
 
54
 
 
0.54
 
 
0.84 (0.76 to 0.92)
 
 
0.37 (0.15 to 0.56)
 
 
 
 
  …1
 
 
6
 
 
100
 
 
77
 
 
0.77
 
 
0.88 (0.79 to 0.97)
 
 
0.04 (0.01 to 0.14)
 
 
 
 
  …2
 
 
7
 
 
100
 
 
55
 
 
0.55
 
 
0.84 (0.69 to 1.00)
 
 
0.30 (0.01 to 0.61)
 
 
 
 
  …3
 
 
9
 
 
75
 
 
80
 
 
0.55
 
 
0.82 (0.69 to 0.96)
 
 
0.18 (0.01 to 0.45)
 
 
 
 
  2
 
 
7
 
 
100
 
 
96
 
 
0.96
 
 
0.99 (0.97 to 1.00)
 
 
0.52 (0.04 to 0.75)
 
 
 
 
  …1
 
 
5
 
 
100
 
 
97
 
 
0.97
 
 
0.99 (0.97 to 1.00)
 
 
0.41 (0.00 to 0.65)
 
 
 
 
  …3
 
 
2
 
 
100
 
 
99
 
 
0.99
 
 
0.99 (0.99 to 1.00)
 
 
0.25 (0.02 to 0.50)
 
 
 
 
  3
 
 
2
 
 
100
 
 
90
 
 
0.90
 
 
0.95 (0.86 to 1.04)
 
 
0.08 (0.00 to 0.33)
 
 
 
 
B
 
 
 
 
  Base
 
 
137
 
 
91
 
 
92
 
 
0.83
 
 
0.96 (0.93 to 0.98)
 
 
0.92 (0.87 to 0.95)
 
 
 
 
  1
 
 
67
 
 
93
 
 
92
 
 
0.84
 
 
0.95 (0.93 to 0.98)
 
 
0.77 (0.66 to 0.85)
 
 
 
 
  …1
 
 
39
 
 
92
 
 
81
 
 
0.74
 
 
0.90 (0.87 to 0.94)
 
 
0.37 (0.25 to 0.52)
 
 
 
 
  …2
 
 
26
 
 
96
 
 
83
 
 
0.79
 
 
0.94 (0.91 to 0.97)
 
 
0.40 (0.22 to 0.54)
 
 
 
 
  …3
 
 
2
 
 
100
 
 
93
 
 
0.93
 
 
0.96 (0.90 to 1.02)
 
 
0.06 (0.01 to 0.23)
 
 
 
 
  2
 
 
38
 
 
74
 
 
86
 
 
0.59
 
 
0.86 (0.80 to 0.92)
 
 
0.40 (0.25 to 0.59)
 
 
 
 
  …1
 
 
20
 
 
85
 
 
85
 
 
0.70
 
 
0.91 (0.85 to 0.97)
 
 
0.37 (0.18 to 0.59)
 
 
 
 
  …2
 
 
16
 
 
81
 
 
90
 
 
0.71
 
 
0.88 (0.77 to 1.00)
 
 
0.35 (0.12 to 0.54)
 
 
 
 
  …3
 
 
2
 
 
100
 
 
77
 
 
0.77
 
 
0.87 (0.68 to 1.07)
 
 
0.03 (0.00 to 0.12)
 
 
 
 
  3
 
 
32
 
 
97
 
 
78
 
 
0.75
 
 
0.92 (0.89 to 0.96)
 
 
0.40 (0.23 to 0.56)
 
 
 
 
  …1
 
 
12
 
 
92
 
 
82
 
 
0.74
 
 
0.90 (0.83 to 0.97)
 
 
0.18 (0.06 to 0.36)
 
 
 
 
  …2
 
 
13
 
 
100
 
 
77
 
 
0.77
 
 
0.92 (0.88 to 0.96)
 
 
0.20 (0.09 to 0.37)
 
 
 
 
  …3
 
 
6
 
 
100
 
 
92
 
 
0.92
 
 
0.96 (0.93 to 0.99)
 
 
0.16 (0.05 to 0.34)
 
 
 
 
C
 
 
 
 
  Base
 
 
47
 
 
89
 
 
85
 
 
0.74
 
 
0.93 (0.89 to 0.97)
 
 
0.73 (0.56 to 0.84)
 
 
 
 
  1
 
 
24
 
 
83
 
 
86
 
 
0.70
 
 
0.90 (0.84 to 0.97)
 
 
0.42 (0.23 to 0.62)
 
 
 
 
  …1
 
 
17
 
 
88
 
 
91
 
 
0.79
 
 
0.93 (0.87 to 0.99)
 
 
0.39 (0.21 to 0.60)
 
 
 
 
  …2
 
 
5
 
 
80
 
 
88
 
 
0.68
 
 
0.86 (0.75 to 0.97)
 
 
0.05 (0.01 to 0.11)
 
 
 
 
  …3
 
 
2
 
 
100
 
 
88
 
 
0.88
 
 
0.93 (0.83 to 1.02)
 
 
0.04 (0.01 to 0.15)
 
 
 
 
  2
 
 
18
 
 
100
 
 
82
 
 
0.82
 
 
0.93 (0.90 to 0.97)
 
 
0.40 (0.17 to 0.54)
 
 
 
 
  …1
 
 
6
 
 
100
 
 
67
 
 
0.67
 
 
0.86 (0.74 to 0.99)
 
 
0.22 (0.00 to 0.44)
 
 
 
 
  …2
 
 
3
 
 
100
 
 
99
 
 
0.99
 
 
0.99 (0.99 to 1.00)
 
 
0.32 (0.05 to 0.65)
 
 
 
 
  …3
 
 
9
 
 
100
 
 
83
 
 
0.83
 
 
0.92 (0.88 to 0.96)
 
 
0.11 (0.05 to 0.22)
 
 
 
 
  3
 
 
5
 
 
100
 
 
95
 
 
0.95
 
 
0.98 (0.97 to 1.00)
 
 
0.29 (0.05 to 0.57)
 
 
 
 
  …1
 
 
3
 
 
100
 
 
90
 
 
0.90
 
 
0.96 (0.90 to 1.03)
 
 
0.16 (0.00 to 0.49)
 
 
 
 
A11 or A12
 
 
13
 
 
90
 
 
68
 
 
0.58
 
 
0.85 (0.75 to 0.96)
 
 
0.24 (0.04 to 0.49)
 
 
 
 
A12 or A13
 
 
16
 
 
100
 
 
50
 
 
0.50
 
 
0.82 (0.72 to 0.93)
 
 
0.35 (0.08 to 0.56)
 
 
 
 
B11 or B12
 
 
28
 
 
96
 
 
87
 
 
0.83
 
 
0.94 (0.92 to 0.97)
 
 
0.43 (0.28 to 0.64)
 
 
 
 
B11 or B12 or B21
 
 
48
 
 
96
 
 
84
 
 
0.80
 
 
0.94 (0.92 to 0.96)
 
 
0.57 (0.42 to 0.72)
 
 
 
 
medial malleolus
 
 
78
 
 
90
 
 
77
 
 
0.67
 
 
0.92 (0.88 to 0.95)
 
 
0.74 (0.63 to 0.81)
 
 
 
 
 
The outcome measures for the most important groups. Criterion based on
Youden’s Index (Youden 1950; Aoki et al. 1997; Shapiro 1999; Greiner et
al. 2000) defined as YI(c)=max_{c}(Se(c)+Sp(c)-1). This is identical
(from an optimization point of view) to the method that maximizes the
sum of Sensitivity and Specificity (Albert and Harris 1987; Zweig and
Campbell 1993) and to the criterion that maximizes concordance, wich is
a monotone function of the AUC
 
 
 
 
 
 
 
Statisic
 
 
Mean
 
 
 
 
 
 
Sensitivity
 
 
0.90
 
 
 
 
Specificity
 
 
0.85
 
 
 
 
YoudenJ
 
 
0.75
 
 
 
 
AUC
 
 
0.93
 
 
 
 
prAUC
 
 
0.63
 
 
 
 
 
Mean weighted summary statistics where each column is multiplied by the
number of cases and then divided by the total. This is applied to for
cases matching
‘^(ao|alt_ao) malleolar|^general (Malleolar|Fracture)$’, total of
40 measurements.
 
 
 
 
 
 Fibula fractures 
 
 
 
 
 
 
Cases (n=409)
 
 
Sensitivity (%)
 
 
Specificity (%)
 
 
Youden’s J
 
 
AUC (95% CI)
 
 
PR-AUC (95% CI)
 
 
 
 
 
 
General
 
 
37
 
 
92
 
 
87
 
 
0.78
 
 
0.93 (0.89 to 0.97)
 
 
0.52 (0.36 to 0.66)
 
 
 
 
2
 
 
5
 
 
80
 
 
85
 
 
0.65
 
 
0.78 (0.52 to 1.04)
 
 
0.04 (0.01 to 0.10)
 
 
 
 
2A
 
 
3
 
 
67
 
 
94
 
 
0.61
 
 
0.72 (0.27 to 1.16)
 
 
0.03 (0.00 to 0.10)
 
 
 
 
2B
 
 
2
 
 
100
 
 
84
 
 
0.84
 
 
0.87 (0.80 to 0.94)
 
 
0.02 (0.01 to 0.05)
 
 
 
 
3
 
 
32
 
 
94
 
 
88
 
 
0.82
 
 
0.94 (0.90 to 0.97)
 
 
0.48 (0.33 to 0.64)
 
 
 
 
3A
 
 
16
 
 
94
 
 
87
 
 
0.80
 
 
0.91 (0.85 to 0.97)
 
 
0.22 (0.11 to 0.39)
 
 
 
 
3B
 
 
16
 
 
88
 
 
89
 
 
0.76
 
 
0.90 (0.81 to 0.99)
 
 
0.38 (0.14 to 0.59)
 
 
 
 
 
The outcome measures for the most important groups. Criterion based on
Youden’s Index (Youden 1950; Aoki et al. 1997; Shapiro 1999; Greiner et
al. 2000) defined as YI(c)=max_{c}(Se(c)+Sp(c)-1). This is identical
(from an optimization point of view) to the method that maximizes the
sum of Sensitivity and Specificity (Albert and Harris 1987; Zweig and
Campbell 1993) and to the criterion that maximizes concordance, wich is
a monotone function of the AUC
 
 
 
 
 
 
 Inter-rater agreements 
 
 ICC 
 Intraclass correlation coefficient (ICC) 
 
 
 
 
 
 
ICC (95% CI)
 
 
 
 
 
 
General
 
 
 
 
Fracture
 
 
 
 
  Malleolar
 
 
0.86 (0.84 - 0.88)
 
 
 
 
Malleolar
 
 
 
 
A
 
 
 
 
  base
 
 
0.84 (0.81 - 0.86)
 
 
 
 
  1
 
 
0.82 (0.79 - 0.84)
 
 
 
 
  …1
 
 
-0.02 (-0.07 - 0.04)
 
 
 
 
  …2
 
 
0.56 (0.51 - 0.61)
 
 
 
 
  …3
 
 
0.71 (0.67 - 0.75)
 
 
 
 
  2
 
 
0.00 (-0.06 - 0.06)
 
 
 
 
  …2
 
 
0.00 (-0.06 - 0.06)
 
 
 
 
  …3
 
 
0.00 (-0.05 - 0.06)
 
 
 
 
  3
 
 
0.00 (-0.05 - 0.06)
 
 
 
 
  …2
 
 
0.00 (-0.05 - 0.06)
 
 
 
 
B
 
 
 
 
  base
 
 
0.89 (0.87 - 0.91)
 
 
 
 
  1
 
 
0.76 (0.72 - 0.79)
 
 
 
 
  …1
 
 
0.50 (0.44 - 0.55)
 
 
 
 
  …2
 
 
0.24 (0.17 - 0.30)
 
 
 
 
  …3
 
 
0.27 (0.20 - 0.33)
 
 
 
 
  2
 
 
0.40 (0.34 - 0.46)
 
 
 
 
  …1
 
 
0.37 (0.30 - 0.43)
 
 
 
 
  …2
 
 
0.20 (0.14 - 0.26)
 
 
 
 
  …3
 
 
0.00 (-0.06 - 0.06)
 
 
 
 
  3
 
 
0.12 (0.06 - 0.18)
 
 
 
 
  …1
 
 
0.14 (0.08 - 0.20)
 
 
 
 
  …3
 
 
0.00 (-0.05 - 0.06)
 
 
 
 
C
 
 
 
 
  base
 
 
0.76 (0.72 - 0.79)
 
 
 
 
  1
 
 
0.63 (0.58 - 0.68)
 
 
 
 
  …1
 
 
0.49 (0.43 - 0.55)
 
 
 
 
  …2
 
 
0.00 (-0.06 - 0.06)
 
 
 
 
  2
 
 
0.47 (0.41 - 0.53)
 
 
 
 
  …1
 
 
0.54 (0.49 - 0.60)
 
 
 
 
  3
 
 
0.00 (-0.06 - 0.06)
 
 
 
 
  …1
 
 
0.00 (-0.05 - 0.06)
 
 
 
 
  …2
 
 
0.00 (-0.05 - 0.06)
 
 
 
 
 
 
 Kappa values for Frank & Max 
 
 
 
 
 
 
ICC (95% CI)
 
 
 
 
 
 
General
 
 
 
 
Fracture
 
 
 
 
  Malleolar
 
 
0.85 (0.80 - 0.91)
 
 
 
 
Malleolar
 
 
 
 
A
 
 
 
 
  base
 
 
0.85 (0.79 - 0.91)
 
 
 
 
  1
 
 
0.82 (0.76 - 0.89)
 
 
 
 
  …1
 
 
-0.02 (-0.03 - -0.01)
 
 
 
 
  …2
 
 
0.70 (0.55 - 0.84)
 
 
 
 
  …3
 
 
0.81 (0.73 - 0.89)
 
 
 
 
  2
 
 
0.00 (-0.01 - 0.00)
 
 
 
 
  …2
 
 
0.00 (-0.01 - 0.00)
 
 
 
 
  …3
 
 
0.00 (0.00 - 0.00)
 
 
 
 
  3
 
 
1.0 (identical)
 
 
 
 
  …2
 
 
1.0 (identical)
 
 
 
 
B
 
 
 
 
  base
 
 
0.91 (0.87 - 0.95)
 
 
 
 
  1
 
 
0.84 (0.78 - 0.90)
 
 
 
 
  …1
 
 
0.66 (0.57 - 0.75)
 
 
 
 
  …2
 
 
0.46 (0.33 - 0.60)
 
 
 
 
  …3
 
 
0.40 (-0.15 - 0.94)
 
 
 
 
  2
 
 
0.53 (0.32 - 0.74)
 
 
 
 
  …1
 
 
0.39 (0.16 - 0.62)
 
 
 
 
  …2
 
 
0.00 (0.00 - 0.00)
 
 
 
 
  …3
 
 
0.00 (0.00 - 0.00)
 
 
 
 
  3
 
 
0.00 (0.00 - 0.00)
 
 
 
 
  …1
 
 
0.00 (0.00 - 0.00)
 
 
 
 
  …3
 
 
1.0 (identical)
 
 
 
 
C
 
 
 
 
  base
 
 
0.78 (0.67 - 0.90)
 
 
 
 
  1
 
 
0.67 (0.52 - 0.82)
 
 
 
 
  …1
 
 
0.69 (0.54 - 0.84)
 
 
 
 
  …2
 
 
0.00 (0.00 - 0.00)
 
 
 
 
  2
 
 
0.49 (0.14 - 0.84)
 
 
 
 
  …1
 
 
0.49 (0.14 - 0.84)
 
 
 
 
  3
 
 
0.00 (0.00 - 0.00)
 
 
 
 
  …1
 
 
0.00 (0.00 - 0.00)
 
 
 
 
  …2
 
 
1.0 (identical)
 
 
 
 
 
Kappa estimates betwen MG & FI
 
 
 
 
 
 
 
 Image analysis 
 Note that we are only looking at that particular category and in some
cases the another category trumps the investigated category, e.g. a
C-type fracture may have an 80% probability of being in B-class and thus
be flagged while the C-category scores 90% percent and theoretically the
network has still picked the correct category. The analysis is though
primarily for investigating the single category and less for making sure
that the correct category has the highest probability, if all categories
have a low probability it doesn’t matter that the correct one was at 10%
while the others were at 1-2 % as we are more interested in the network
flagging the correct category. 
 
 Malloelar 
 
 A-class 
 
 Correct true positive 
   
 
 
 
 
 
 
Options (%)
 
 
 
 
 
 
 
 
 
 
Row no.
 
 
A
 
 
B
 
 
C
 
 
 
 
 
 
Max
 
 
True categories
 
 
 
 
 
 
1
 
 
100
 
 
2
 
 
1
 
 
 
 
 
 
A
 
 
A12
 
 
 
 
2
 
 
100
 
 
3
 
 
1
 
 
 
 
 
 
A
 
 
A12
 
 
 
 
3
 
 
98
 
 
21
 
 
5
 
 
 
 
 
 
A
 
 
A13
 
 
 
 
4
 
 
98
 
 
3
 
 
9
 
 
 
 
 
 
A
 
 
A12
 
 
 
 
 
Correctly classified cases as  ao_malleolar_A  sorted from highest
probability to lowest
 
 
 
 
 
 Failed true positive 
   
 
 
 
 
 
 
Options (%)
 
 
 
 
 
 
 
 
 
 
Row no.
 
 
A
 
 
B
 
 
C
 
 
 
 
 
 
Max
 
 
True categories
 
 
 
 
 
 
1
 
 
1
 
 
86
 
 
21
 
 
 
 
 
 
B
 
 
A13
 
 
 
 
2
 
 
2
 
 
5
 
 
89
 
 
 
 
 
 
C
 
 
A13
 
 
 
 
3
 
 
2
 
 
2
 
 
37
 
 
 
 
 
 
C
 
 
A13
 
 
 
 
4
 
 
3
 
 
4
 
 
5
 
 
 
 
 
 
C
 
 
A13
 
 
 
 
 
Incorrectly classified cases where the network failed to detect as
 ao_malleolar_A , sorted from lowest probability to highest.
 
 
 
 
 
 Failed true negative 
   
 
 
 
 
 
 
Options (%)
 
 
 
 
 
 
 
 
 
 
Row no.
 
 
A
 
 
B
 
 
C
 
 
 
 
 
 
Max
 
 
True categories
 
 
 
 
 
 
1
 
 
98
 
 
67
 
 
22
 
 
 
 
 
 
A
 
 
B11
 
 
 
 
2
 
 
92
 
 
5
 
 
14
 
 
 
 
 
 
A
 
 
 
 
 
 
3
 
 
92
 
 
46
 
 
13
 
 
 
 
 
 
A
 
 
B12
 
 
 
 
4
 
 
83
 
 
9
 
 
19
 
 
 
 
 
 
A
 
 
 
 
 
 
 
Incorrectly classified cases where the network wrongly detected as
 ao_malleolar_A , sorted from highest probability to lowest.
 
 
 
 
 
 
 B-class 
 
 Correct true positive 
   
 
 
 
 
 
 
Options (%)
 
 
 
 
 
 
 
 
 
 
Row no.
 
 
A
 
 
B
 
 
C
 
 
 
 
 
 
Max
 
 
True categories
 
 
 
 
 
 
1
 
 
0
 
 
100
 
 
4
 
 
 
 
 
 
B
 
 
B11
 
 
 
 
2
 
 
1
 
 
100
 
 
4
 
 
 
 
 
 
B
 
 
B11
 
 
 
 
3
 
 
2
 
 
100
 
 
6
 
 
 
 
 
 
B
 
 
B11
 
 
 
 
4
 
 
1
 
 
100
 
 
9
 
 
 
 
 
 
B
 
 
B11
 
 
 
 
 
Correctly classified cases as  ao_malleolar_B  sorted from highest
probability to lowest
 
 
 
 
 
 Failed true positive 
   
 
 
 
 
 
 
Options (%)
 
 
 
 
 
 
 
 
 
 
Row no.
 
 
A
 
 
B
 
 
C
 
 
 
 
 
 
Max
 
 
True categories
 
 
 
 
 
 
1
 
 
25
 
 
4
 
 
74
 
 
 
 
 
 
C
 
 
B13
 
 
 
 
2
 
 
4
 
 
5
 
 
70
 
 
 
 
 
 
C
 
 
B11
 
 
 
 
3
 
 
22
 
 
5
 
 
52
 
 
 
 
 
 
C
 
 
B12
 
 
 
 
4
 
 
6
 
 
5
 
 
9
 
 
 
 
 
 
C
 
 
B11
 
 
 
 
 
Incorrectly classified cases where the network failed to detect as
 ao_malleolar_B , sorted from lowest probability to highest.
 
 
 
 
 
 Failed true negative 
   
 
 
 
 
 
 
Options (%)
 
 
 
 
 
 
 
 
 
 
Row no.
 
 
A
 
 
B
 
 
C
 
 
 
 
 
 
Max
 
 
True categories
 
 
 
 
 
 
1
 
 
3
 
 
97
 
 
5
 
 
 
 
 
 
B
 
 
C11
 
 
 
 
2
 
 
21
 
 
95
 
 
20
 
 
 
 
 
 
B
 
 
A13
 
 
 
 
3
 
 
1
 
 
94
 
 
1
 
 
 
 
 
 
B
 
 
 
 
 
 
4
 
 
43
 
 
93
 
 
3
 
 
 
 
 
 
B
 
 
A12
 
 
 
 
 
Incorrectly classified cases where the network wrongly detected as
 ao_malleolar_B , sorted from highest probability to lowest.
 
 
 
 
 
 
 C-class 
 
 Correct true positive 
   
 
 
 
 
 
 
Options (%)
 
 
 
 
 
 
 
 
 
 
Row no.
 
 
A
 
 
B
 
 
C
 
 
 
 
 
 
Max
 
 
True categories
 
 
 
 
 
 
1
 
 
0
 
 
1
 
 
100
 
 
 
 
 
 
C
 
 
C21
 
 
 
 
2
 
 
0
 
 
0
 
 
100
 
 
 
 
 
 
C
 
 
C11
 
 
 
 
3
 
 
0
 
 
15
 
 
100
 
 
 
 
 
 
C
 
 
C11
 
 
 
 
4
 
 
0
 
 
11
 
 
100
 
 
 
 
 
 
C
 
 
C11
 
 
 
 
 
Correctly classified cases as  ao_malleolar_C  sorted from highest
probability to lowest
 
 
 
 
 
 Failed true positive 
   
 
 
 
 
 
 
Options (%)
 
 
 
 
 
 
 
 
 
 
Row no.
 
 
A
 
 
B
 
 
C
 
 
 
 
 
 
Max
 
 
True categories
 
 
 
 
 
 
1
 
 
3
 
 
97
 
 
5
 
 
 
 
 
 
B
 
 
C11
 
 
 
 
2
 
 
6
 
 
61
 
 
6
 
 
 
 
 
 
B
 
 
C11
 
 
 
 
3
 
 
11
 
 
16
 
 
11
 
 
 
 
 
 
B
 
 
C21
 
 
 
 
4
 
 
22
 
 
4
 
 
17
 
 
 
 
 
 
A
 
 
C31
 
 
 
 
 
Incorrectly classified cases where the network failed to detect as
 ao_malleolar_C , sorted from lowest probability to highest.
 
 
 
 
 
 Failed true negative 
   
 
 
 
 
 
 
Options (%)
 
 
 
 
 
 
 
 
 
 
Row no.
 
 
A
 
 
B
 
 
C
 
 
 
 
 
 
Max
 
 
True categories
 
 
 
 
 
 
1
 
 
2
 
 
2
 
 
100
 
 
 
 
 
 
C
 
 
 
 
 
 
2
 
 
0
 
 
53
 
 
99
 
 
 
 
 
 
C
 
 
B11
 
 
 
 
3
 
 
0
 
 
87
 
 
99
 
 
 
 
 
 
C
 
 
B11
 
 
 
 
4
 
 
1
 
 
71
 
 
98
 
 
 
 
 
 
C
 
 
B11
 
 
 
 
 
Incorrectly classified cases where the network wrongly detected as
 ao_malleolar_C , sorted from highest probability to lowest.
 
 
 
 
 
 
 


 

 

 

 

 


 
 

 
 
